# Proteomic Analysis of Lipid Droplets from Caco-2/TC7 Enterocytes Identifies Novel Modulators of Lipid Secretion

**DOI:** 10.1371/journal.pone.0053017

**Published:** 2013-01-02

**Authors:** Frauke Beilstein, Julien Bouchoux, Monique Rousset, Sylvie Demignot

**Affiliations:** 1 Université Pierre et Marie Curie, UMR S 872, Les Cordeliers, Paris, France; 2 Inserm, U 872, Paris, France; 3 Ecole Pratique des Hautes Etudes, Laboratoire de Pharmacologie Cellulaire et Moléculaire, Paris, France; 4 Université Paris Descartes, UMR S 872, Paris, France; 5 Institut de Cardiométabolisme et Nutrition (ICAN), Paris, France; Fundação Oswaldo Cruz, Brazil

## Abstract

In enterocytes, the dynamic accumulation and depletion of triacylglycerol (TAG) in lipid droplets (LD) during fat absorption suggests that cytosolic LD-associated TAG contribute to TAG-rich lipoprotein (TRL) production. To get insight into the mechanisms controlling the storage/secretion balance of TAG, we used as a tool hepatitis C virus core protein, which localizes onto LDs, and thus may modify their protein coat and decrease TRL secretion. We compared the proteome of LD fractions isolated from Caco-2/TC7 enterocytes expressing or not hepatitis C virus core protein by a differential proteomic approach (isobaric tag for relative and absolute quantitation (iTRAQ) labeling coupled with liquid chromatography and tandem mass spectrometry). We identified 42 proteins, 21 being involved in lipid metabolism. Perilipin-2/ADRP, which is suggested to stabilize long term-stored TAG, was enriched in LD fractions isolated from Caco-2/TC7 expressing core protein while perilipin-3/TIP47, which is involved in LD synthesis from newly synthesized TAG, was decreased. Endoplasmic reticulum-associated proteins were strongly decreased, suggesting reduced interactions between LD and endoplasmic reticulum, where TRL assembly occurs. For the first time, we show that 17β-hydroxysteroid dehydrogenase 2 (DHB2), which catalyzes the conversion of 17-keto to 17 β-hydroxysteroids and which was the most highly enriched protein in core expressing cells, is localized to LD and interferes with TAG secretion, probably through its capacity to inactivate testosterone. Overall, we identified potential new players of lipid droplet dynamics, which may be involved in the balance between lipid storage and secretion, and may be altered in enterocytes in pathological conditions such as insulin resistance, type II diabetes and obesity.

## Introduction

Lipid droplets (LD) comprise a core of triacylglycerols (TAG) and cholesterol esters surrounded by a monolayer of phospholipids, cholesterol and of a variety of proteins [1], [2]. TAG synthesis takes place at the endoplasmic reticulum (ER) membrane, where enzymes required for their synthesis are located. It is now widely accepted that the newly synthesized TAG accumulate between the two phospholipid leaflets of the ER membrane and that, after reaching a critical size, the nascent lipid droplet may bud off toward the cytosol but also, in hepatocytes and enterocytes, into the ER lumen where triglyceride-rich lipoprotein (TRL) assembly occurs [1], [3], [4]. The current model of TRL assembly proposes a two-step process, consisting of the formation of a lipid-poor apolipoprotein B (apoB) particle followed by its fusion with a luminal TG-rich apoB-free lipid droplet formed independently. The microsomal TAG transfer protein (MTP) plays an essential role in TRL assembly, for the co-translational lipid recruitment by apoB to form the primordial apoB particle as well as for the luminal LD production (for reviews, see [5], [6]).

The function and fate of TAG present in LD vary depending on cell types. LD were essentially studied in adipocytes, because they are specialized in TAG storage and have a single very large lipid droplet filling the cytoplasm. Upon fasting, TAG of the LD are hydrolyzed and fatty acids are released into the circulation to provide energy to other organs such as muscles and heart. In mammary cells, the LD are exocytosed to create the milk globules during lactation. In hepatocytes and enterocytes, TAG present in cytosolic LD may contribute to TRL assembly through a mechanism of hydrolysis–reesterification [7], [8]. The fatty acids, mono- and diacylglycerols released by lipolysis from cytosolic LD can participate to new TAG synthesis at the ER membrane. However, the proteins and enzymes involved in the control of the TAG partition between cytosol and ER lumen, i.e. between storage and secretion, and the underlying mechanisms, are still poorly understood in these cells.

The proteins associated with LD have been characterized in different specialized mammalian cell types including 3T3-L1 adipocytes, mammary epithelial cells, hepatic cells (for review [9]), Caco-2/TC7 enterocytes [10], muscle cells [11] and insulin-producing β-cells [12]. These studies indicate that the proteome of cytosolic LD depends on the cell type although common features occur. For example, the structural PLIN proteins (previously known as PAT family proteins) [13] are always identified on LD. Perilipin-1 is found specifically on the adipocyte lipid droplet, perilipin-5/OXPAT is expressed in cells that have a high capacity for fatty acid oxidation, such as cardiac muscle cells, while perilipin-2/ADFP/ADRP and perilipin-3/TIP47 are ubiquitous (for review [14]). Similarly, proteins involved in lipid metabolism, intracellular traffic or signalling are always identified, but can vary from one cell type to another [9]. Moreover, the protein composition of LD in a given cell type may differ depending on the physiopathological state of the cell. In summary, although cytosolic lipid droplets were previously considered simply as long term lipid storage bodies, it is now clear that they are cellular organelles involved actively in the control of lipid metabolism, in direct and dynamic interaction with other organelles like the ER and mitochondria [11], [12], [15], [16].

Observations made in enterocytes *in vivo* during lipid absorption have clearly shown that a dynamic accumulation and depletion of TAG in LD occurs during the process of fat absorption, suggesting that TAG present in cytosolic LD contribute to chylomicron production [10], [17], [18]. Recently, we characterized the protein endowment of cytosolic LD isolated from Caco-2/TC7 enterocytes 24 h after incubation with lipid micelles and thus in a state of cytosolic LD-associated TAG mobilization [10]. When supplied with lipid micelles, these human enterocytes are able to produce TRL and to store, in cytosolic LD, TAG that can be subsequently mobilized to contribute to TRL production in the absence of lipid micelles [19], [20]. Furthermore, we showed that the extent of TAG targeting into the ER lumen, and thus the balance between storage and secretion, is modulated by nutrients, including glucose [19] or polyphenols [21]. High levels of intestinally derived lipoproteins are associated with increased cardiovascular risk and there is evidence of altered TRL secretion by intestine in pathological conditions, such as insulin resistance, type II diabetes and obesity [22], [23], [24], [25]. An imbalance between the cytosolic and luminal LD dynamics could contribute to this altered TRL secretion and it is thus important to determine the underlying mechanisms that control the TAG partition between cytosol and ER in enterocytes.

The hepatitis C virus (HCV) core protein has the ability to impair the balance between TAG storage and secretion in hepatocytes [26]. This structural protein, forming the capsid shell of HCV, is targeted to the cytosolic side of the ER membrane from where it migrates onto the surface of LD, possibly by lateral diffusion [27]. Infected patients can develop hypobetalipoproteinemia as well as liver steatosis [26], [28] and studies in transgenic mice expressing HCV core protein indicated that core protein on its own is sufficient to provoke these effects in hepatocytes, i.e. a decrease of TRL secretion and a cytosolic accumulation of LD [29], [30]. Moreover, although lipoprotein secretion was not examined, it has been shown that cells transfected with HCV core protein accumulate LD [31], [32].

This study aimed to identify, in Caco-2/TC7 enterocytes, LD-associated proteins that could be involved in the partition of TAG between storage and secretion. HCV core protein was used as a tool to modify the protein coat of LD. Results demonstrated that HCV core protein expression in Caco-2/TC7 enterocytes impaired their TRL secretion capacity, as compared to control cells. Differential proteomics allowed the identification of proteins that were differentially expressed in LD fractions isolated from Caco-2/TC7 cells expressing HCV core protein or not. Among them, we show for the first time that 17β-hydroxysteroid dehydrogenase 2 (DHB2), a member of the short chain dehydrogenase/reductase superfamily, modulates lipid secretion by Caco-2/TC7 enterocytes.

## Materials and Methods

### Antibodies

Antibodies against calnexin (610524) and PDI were obtained from BD Biosciences; anti-HSP60, anti-GRP78 and anti-HCV core protein (C7-50) antibodies were from Abcam. Anti-HSD17B2 (SAB1400130), anti-LPCAT2 (SAB1401734), anti-3BHS1 (WH0003283M1) and anti-myc (9E10) antibodies were from Sigma. The antibody against AT1B1 (Na+/K+ ATPase α1) (clone C464.6) was from Upstate. The anti-human apoB antibody (1D1) was obtained from the Heart Institute of the University of Ottawa (Canada), the anti-UBXD8 (B01P) antibody was obtained from Abnova, and the anti-GFP antibody was from Roche Applied Science. The sheep anti-perilipin-2 antibody was generously provided by J. McLauchlan [33]. Secondary antibodies were appropriate Alexa Fluor 568-conjugated antibodies and horseradish peroxidase-conjugated IgGs (GE Healthcare UK).

### Plasmids

To allow gene expression in differentiated Caco-2/TC7 cells, the CMV promoter of pEGFP-C1 (BD Bioscience Clonetech) was changed for the SV40 promoter of pGL3 (Promega) to obtain pNeoSV40-EGFP-C1 (provided by M. Le Gall [34]). The HCV core protein serotype 1b, followed by the signal sequence of the envelope protein E1, was obtained from pRSV (provided by N. Pavio [35]) as a HindIII – BamHI PCR Fragment (PC1Hin CCAACCAAGCTTATATGAGCACGAATCCT PC2Bam TTCGGATCCTTAAGCGGAAGCTGGGAT). The resulting PCR fragment (CP) was cloned between the HindIII and BamHI restriction sites of pNeoSV40-EGFP-C1 to yield pGFP-CP.

The vector pCDNA3.1-mycSV40 was made by cloning the SV40 Promotor, isolated as a MluI-HindIII fragment from pGL3 (Promega), between the MluI and HindIII restriction sites of pCDNA3.1-myc (provided by D. Pasdeloup [36]). All the other constructs used were generated by PCR, as described in Table 1, and cloned into pCDNA3.1-mycSV40.

**Table 1:** PCR primers used to generate DNA constructs.

Construct	(residues)	Template	PCR primer
myc-ACSL3	(444–2606)	IMAGE:5226905	F: TTGGAATTCTATGAATAACCACGTGTCTTCAAAACCATCTACC*
			R: GAAGCGGCCGCTTATTTTCTTCCATACATTCGCTCAATGTCCGC
myc-C2043	(54–1031)	IMAGE:3611020	F: TGGGATATCATGGACTCAGAACTCAAGGAAGAAATTCCTGTG
			R: CTCGCGGCCGCTTACATTTTGGACAAGTCATCCTTTAGGGAGTCAG
myc-HSD17B2	(86–1249)	IMAGE:4077164	F: GTCGAATTCAATGAGCACTTTCTTCTCGGACACAGC*
			R: GCTGCGGCCGCCTAGGTGGCCTTTTTCTTGTAGTTAGGCATTCTTAG
myc-LPCAT2	(141–1775)	IMAGE:3347690	F: GGCGATATCATGAGCCGGTGCGCCC
			R: ATATCTAGATCAGTCATCTTTTTTGTCTGAGGTACTCTCTTCATG
myc-HSD3B1	(88–1209)	IMAGE:4755300	F: GCTGAATTCGATGGCCATGACGGGCTGG*
			R: TCAGCGGCCGCTCACTGAGTCTTGGACTTCAGGTTCTC

The PCR primers are listed in pairs, with the forward primer (F) listed first and the reverse primer (R) second. All PCR products were cloned into pCDNA3.1-mycSV40, and the restriction enzyme sites used for cloning are underlined.

* indicates that these primers have a +1 nucleotide after the restriction site to put the gene in frame with the tag.

### Cell Culture, Transfections and Incubations with Lipids and Steroids

We used the human enterocyte-like Caco-2/TC7 cell line, a clone that we derived from the parental Caco-2 cell line [37]. For studies on differentiated Caco-2/TC7 cells, i.e. cells able to secrete TRL, cells were cultured for 17–18 days on semipermeable filters as described previously [10]. When indicated, lipid micelles [sodium taurocholate (2 mM), oleic acid (0.6 mM), lysophosphatidylcholine (0.6 mM), cholesterol (0.05 mM) and 1-O-octadecyl-rac-glycerol (0.2 mM), a stable analogue of 2-monoacylglycerol] were prepared as described previously [19], and added to the upper compartment for the last 24 h of culture. When appropriate, lipid micelles were supplemented with 1 µCi [1-^14^C]oleic acid (Perkin-Elmer Life Sciences) per ml of final medium, as described previously [20]. In some experiments, cells were incubated with 0.1 µM estradiol (E2, Sigma), 0.1 µM testosterone (T, Sigma) or 1 µCi/ml of [6,7-^3^H(N)] estradiol (E2) (Perkin Elmer Life Sciences) in both compartments.

Caco-2/TC7 cells grown to 70% confluence on 35-mm dishes were transfected with 0.5 µg of plasmid DNAs using Lipofectamine 2000 (Invitrogen) according to the manufacturer’s instructions. To obtain the stable Caco-2/TC7 cell lines expressing GFP-HCV core protein (Caco-2/TC7 GFP-CP), transfected cells were sorted by FACS and grown under antibiotic selection.

For immunofluorescence studies, Caco-2/TC7 cells were seeded on glass coverslips and transfected as described above when 60% confluent. When appropriate, five h after transfection, cells were incubated with 0.6 mM oleic acid for the last 24 h of culture to promote lipid droplet formation. Since lipid micelles are cytotoxic to undifferentiated Caco-2/TC7 cells, oleic acid was supplied as complexed to BSA. For this, oleic acid (6 µl from a 100 mM stock solution in chloroform/methanol 2∶1 (v/v) per ml of final medium to prepare) was dried under a stream of nitrogen then complexed to BSA by incubation with fetal calf serum (0.2 ml per ml of final medium to prepare) for 1 h at 37°C. The mixture was then adjusted to 1 ml with culture medium without serum and supplied to the cells.

HEK 293T cells (American Type Tissue Culture Collection) were grown at 37°C in Dulbecco’s modified Eagle medium (DMEM, Invitrogen) supplemented with 10% fetal calf serum.

### shRNA

The use of lentiviral vectors expressing small hairpin RNA (shRNA) was described previously [38]. Briefly, HEK 293T cells were cotransfected with three plasmids: pVSV-G, pCMVDR8.91 (provided by D. Trono [http://tronolab.com/index.php]), and pLKO.1Puro-shDHB2. The cell supernatant containing recombinant lentivirus was harvested 3 days posttransfection and used to transduce Caco-2/TC7 cells, seeded on filters 4 days before, in the presence of hexadimethrine bromide (5 µg/ml polybrene; Sigma). After overnight incubation, the cells were maintained in selective medium containing puromycin (10 µg/ml) for 3 days until confluence then cultured up to day 18 for differentiation. Silencing of HSD17B2 was done by using the 19-nucleotides sequence TGGTGAATGTCAGCAGCAT (shDHB2). ShControl corresponds to a sequence specific to the luciferase gene (GTGCGTTGCTAGTACCAAC). Silencing efficiency was estimated by quantitative RT-PCR.

### Reverse Transcription and Real-time PCR Analysis

Total RNA was isolated using TRI Reagent (Molecular Research Centre) according to the manufacturer’s protocol. The reverse transcription experiments were performed with 1 µg of total RNA in a total volume of 20 µl. PCR reactions were performed in quadruplicate using a Light-cycler machine (Roche). For each reaction, a 1∶400 final dilution of the reverse transcription product was used with 0.4 µM final concentration of each primer in SYBR Green I master mixture (Roche). PCR conditions were one step of denaturation (8 min at 95°C) followed by 45 cycles (each cycle consisted of 10 s at 95°C, 10 s at 60°C (62°C for ACSL3), and 10 s at 72°C). Gene expression was normalized to expression of human ribosomal protein L19. The oligonucleotide primers used for RT-PCR analysis are shown in table S1.

### Fluorescence Microscopy

Cells on glass coverslips were fixed with 4% paraformaldehyde for 10 min at room temperature, and, after two washes with phosphate-buffered saline (PBS), permeabilized with 0.03% saponin in PBS for 30 min. After incubation with the appropriate primary antibody for 1 h at room temperature, the coverslips were washed twice with PBS and incubated with the secondary antibody for an additional h. After two further washes with PBS, they were stained for neutral lipids by incubation for 10 min with BODIPY 493/503 (10 µg/ml; Invitrogen) or with LD540 (0.5 µg/ml), kindly provided by C. Thiele [39], or mounted directly in Fluoprep (BioMérieux) containing 1 µg/ml 4′,6-diamidino-2-phenylindole dihydrochloride (DAPI; Sigma). The samples were examined using a Zeiss LSM 710 Meta confocal microscope.

### Subcellular Fractionation

Lipid droplets from Caco-2/TC7 cells were isolated by density gradient centrifugation as described previously [10]. Briefly, differentiated Caco-2/TC7 cells incubated for 24 h with lipid micelles to promote LD formation were lysed twice using a cell disruption bomb then cell homogenates were centrifuged for 10 min at 1000 g at 15°C. The LD-containing supernatant was adjusted to 0.33 M sucrose, put in a new centrifuge tube, and overlaid with buffers containing sucrose to form a discontinuous sucrose gradient ranging from 0.33 to 0 M. Tubes were centrifuged for 2 h (150 000 *g*, 15°C) and 1 ml fractions were recovered from top to bottom. The pellet was resuspended in 2 ml of buffer.

### Western Blotting

Proteins were resolved on 10% (5% for apoB) sodium dodecyl sulfate-polyacrylamide gels and transferred to Hybond ECL membrane (Amersham). Blots were blocked for 30 min with 5% dried milk powder in 20 mM Tris-HCl, pH 7.6, 137 mM NaCl, and 0.1% Tween 20 (TBS-Tween) and incubated overnight at 4°C with appropriate antibodies diluted in TBS-Tween containing 1% dried milk. Blots were developed by enhanced chemiluminescence using ECL reagent (Amersham) and bands were visualized using the Image Reader LAS-4000 (Fujifilm).

### In-gel Trypsin Digestion, iTRAQ Labelling and Nano-liquid Chromatography-tandem Mass Spectrometry Analysis (LC-MS/MS)

The 1 ml top fractions recovered from Caco-2/TC7 and Caco-2/TC7 GFP-CP cell samples by density gradient ultracentrifugation were freeze-dried, and all of the material was subjected to in-gel trypsin digestion as described previously [10]. The iTRAQ (isobaric tag for relative and absolute quantitation) labelling of peptides was performed according to the manufactureŕs instructions (Applied Biosystems). Briefly, one unit of label (defined as the amount of reagent required to label 100 µg of protein) was thawed, reconstituted in 700 µl of ethanol and incubated with the samples for 2 h at RT. After labelling with different iTRAQ reagents, samples prepared from Caco-2/TC7 and Caco-2/TC7 GFP-CP cells were pooled by pair. Nano-liquid chromatography and tandem mass spectrometry were performed as described in [40]. Four independent experiments were performed in duplicate. The proteins identified in every sample were used to normalize the iTRAQ ratios between the different experiments. The mean value of the iTRAQ ratios for all these “standard” proteins was 0.9945, i.e. very close to one, as expected. To be listed in Table 2, a protein had to be identified at least three times out of the four experiments. For each protein, the normalized iTRAQ ratios and the mean ± SD were calculated, and compared to the theoretical mean (0.9945) to determine whether the protein was significantly differentially expressed in the LD fractions isolated from Caco-2/TC7 GFP-CP cells and Caco-2/TC7 cells.

**Table 2:** List of proteins identified in lipid droplet fractions isolated from Caco-2/TC7 GFP-CP cells compared to that of Caco-2/TC7 cells, ranked by decreasing iTRAQ labelling ratios.

Gene	Swiss-prot	Protein Description	Function	Ratio	s.d.	P<	
HSD17B2	DHB2_HUMAN	Estradiol 17-beta-dehydrogenase 2	Sterol metab.	1.837	0.092	1E-05	*
HSD3B1	3BHS1_HUMAN	3 beta-hydroxysteroid dehydrogenase	Sterol metab.	1.795	0.905	0.12	
MGLL	MGLL_HUMAN	Monoglyceride lipase	Acylglycerol metab.	1.731	0.216	0.0016	*
PLIN2	PLIN2_HUMAN	Perilipin-2/ADRP	PLIN family	1.663	0.255	4E-04	*
LPCAT2	PCAT2_HUMAN	Lysophosphatidylcholine acyltransferase 2	Phospholipid metab.	1.366	0.329	0.04	*
RABA	RAB7A_HUMAN	Ras-related protein Rab-7A	Traffic	1.325	0.398	0.14	
FAF2	FAF2_HUMAN	FAS-associated factor 2, UBXD8	ER stress response	1.302	0.149	0.0039	*
UBXN4	UBXN4_HUMAN	UBX domain-containing protein 4	ER stress response	1.298	0.237	0.16	
SCCPDH	SCPDH_HUMAN	Probable saccharopine dehydrogenase	unknown	1.25	0.21	0.018	*
CYB5R3	NB5R3_HUMAN	NADH-cytochrome b5 reductase 3	Sterol metab.	1.242	0.164	0.0073	*
TUBA1A	TBA1A_HUMAN	Tubulin alpha-1A chain	Microtubules	1.185	0.345	0.44	
C2orf43	CB043_HUMAN	UPF0554 protein C2orf43	unknown	1.172	0.195	0.077	
HSD17B11	DHB11_HUMAN	Estradiol 17-beta-dehydrogenase 11	Sterol metab.	1.101	0.139	0.089	
RAB6A	RAB6A_HUMAN	Ras-related protein Rab-6A	Traffic	1.097	0.398	0.56	
ACSL3	ACSL3_HUMAN	Long-chain-fatty-acid CoA ligase 3	Fatty acid metab.	1.072	0.262	0.47	
RAB5C	RAB5C_HUMAN	Ras-related protein Rab-5C	Traffic	1.065	0.217	0.51	
ATP1A1	AT1B1_HUMAN	Sodium/potassium-transporting ATPase subunit alpha-1	Transporter	1.05	0.797	0.91	
AIFM2	AIFM2_HUMAN	Apoptosis-inducing factor 2	Apoptosis	1.046	0.21	0.54	
HSD17B7	DHB7_HUMAN	3keto-steroid reductase	Sterol metab.	1.006	0.318	0.94	
RAB14	RAB14_HUMAN	Ras-related protein Rab-14	Traffic	0.998	0.372	0.99	
LSS	ERG7_HUMAN	Lanosterol synthase	Sterol metab.	0.997	0.215	0.98	
RAB10	RAB10_HUMAN	Ras-related protein Rab-10	Traffic	0.968	0.37	0.9	
RAB1A	RAB1A_HUMAN	Ras-related protein Rab-1A	Traffic	0.964	0.541	0.9	
DHRS1	DHRS1_HUMAN	Dehydrogenase/reductase SDR family member 1	Lipid metab.	0.956	0.222	0.13	
NSDHL	NSDHL_HUMAN	Sterol-4-alpha-carboxylate 3-dehydrogenase, decarboxylating	Sterol metab.	0.938	0.19	0.46	
DHRS3	DHRS3_HUMAN	Short-chain dehydrogenase/reductase 3	Sterol metab.	0.892	0.325	0.52	
RDH10	RDH10_HUMAN	Retinol dehydrogenase 10	Lipid metab.	0.866	0.123	0.08	
METTL7B	MET7B_HUMAN	Methyltransferase-like protein 7B	unknown	0.801	0.131	0.008	*
ACSL5	ACSL5_HUMAN	Long-chain-fatty-acid–CoA ligase 5	Fatty acid metab.	0.79	0.3	0.36	
SQLE	ERG1_HUMAN	Squalene monooxygenase	Sterol metab.	0.739	0.168	0.12	
PLIN3	PLIN3_HUMAN	Perilipin-3/TIP47	PLIN family	0.715	0.281	0.039	*
CANX	CALX_HUMAN	Calnexin	Chaperon	0.711	0.276	0.083	
ERO1L	ERO1A_HUMAN	ERO1-like protein alpha	Oxidoreductase	0.702	0.252	0.061	
ATP1B1	AT1A1_HUMAN	Sodium/potassium-transporting ATPase subunit beta-1	Transporter	0.699	0.399	0.24	
METTL7A	MET7A_HUMAN	Methyltransferase-like protein 7A	unknown	0.692	0.163	0.0061	*
DDOST	OST48_HUMAN	Dolichyl-diphosphooligosac.-prot.glycosyltransferase 48 kDa sub.	Protein glycosylation	0.566	0.355	0.17	
P4HB	PDIA1_HUMAN	Protein disulfide-isomerase	Lipoprotein metab.	0.557	0.171	5E-04	*
HSP90B	ENPL_HUMAN	Endoplasmin, Heat shock protein 90 kDa beta member 1	Chaperon	0.555	0.108	2E-04	*
PDIA6	PDIA6_HUMAN	Protein disulfide-isomerase 6	Chaperon	0.539	0.084	0.0017	*
PDIA3	PDIA3_HUMAN	Protein disulfide-isomerase 3	Chaperon	0.511	0.118	0.019	*
HSPA5	GRP78_HUMAN	78 kDa glucose-regulated protein	Chaperon	0.507	0.165	0.036	*
MTTP	MTP_HUMAN	Microsomal triglyceride transfer proteinlarge subunit	Lipoprotein metab.	0.389	0.233	0.014	*

The two samples of peptides generated by trypsin digestion of the proteins present in lipid droplets fractions were labelled with two different iTRAQ labels then analyzed by LC-MS/MS. The proteins listed were identified in at least three out of four independent experiments performed in duplicate. An iTRAQ ratio above one indicates that this protein was more abundant in the lipid droplet fraction of Caco-2/TC7 GFP-CP cells than in that isolated from Caco-2/TC7 cells. Conversely, a ratio below one indicates that this protein is less abundant in the lipid droplet fraction of Caco-2/TC7 GFP-CP cells than in that isolated from Caco-2/TC7 cells. Stars highlight proteins whose amounts are significantly different between the two cell lines (P<0.05).

### Lipid Analysis and Estradiol/Estrone Analysis

After incubation with lipid micelles containing [1-^14^C]oleic acid, lipids extracted from cells and culture media were analyzed as described previously [20]. Briefly, lipids were extracted with chloroform/methanol (2∶1, v/v) and fractionated by TLC. Incorporation of [1-^14^C]oleic acid into lipids was measured by liquid-scintillation counting of excised radioactive bands of the TLC plates.

The DHB2 activity assay was performed using a protocol adapted from [41]. Basolateral media (0.5 ml) of cells incubated with [^3^H]E2 was extracted with 1 ml ethyl acetate: isooctane (1∶1, v/v) then the organic phase was evaporated. The residue was dissolved with 50 µl chloroform/methanol (2∶1, v/v) and mixed with carrier steroids (250 nmoles of E1 and E2 each). Steroids were separated by TLC using chloroform: ethyl acetate (3∶1) as the mobile phase and visualized with I_2_ vapour. The E1 and E2 spots were excised and measured by liquid-scintillation counting.

### Statistical Analysis

Data are presented as means ± SD. Statistical significance was evaluated using Student’s *t* test for unpaired data.

## Results

### Creation of the HCV Core Protein-expressing Cell Line Caco-2/TC7 GFP-CP

To study the effect of HCV core protein on TAG balance between storage and secretion in Caco-2/TC7 enterocytes, we generated the cell line Caco-2/TC7 GFP-CP that expresses the HCV core protein. For this, the core gene followed by the signal sequence of the next protein of the polyprotein, i. e. envelope protein E1, from HCV genotype 1b was cloned into the GFP expression vector pNeoSV40-EGFP-C1. The signal sequence targets the core protein to the ER membrane and, after cleavage by signal peptidase and signal peptide peptidase, core protein will traffic to lipid droplets [27], [42]. An N-terminal fusion was thus required in order to prevent core protein separation from the GFP-tag after cleavage of the signal peptide. Expression of the GFP-CP fusion protein was under the control of the SV40 promoter, which has been shown to preserve moderate expression of the transgene even in differentiated Caco-2/TC7 cells [34]. GFP positive cells were sorted by FACS and, after antibiotic selection, several clones were isolated and the stable cell line Caco-2/TC7 GFP-CP was established.

**Figure 1:**
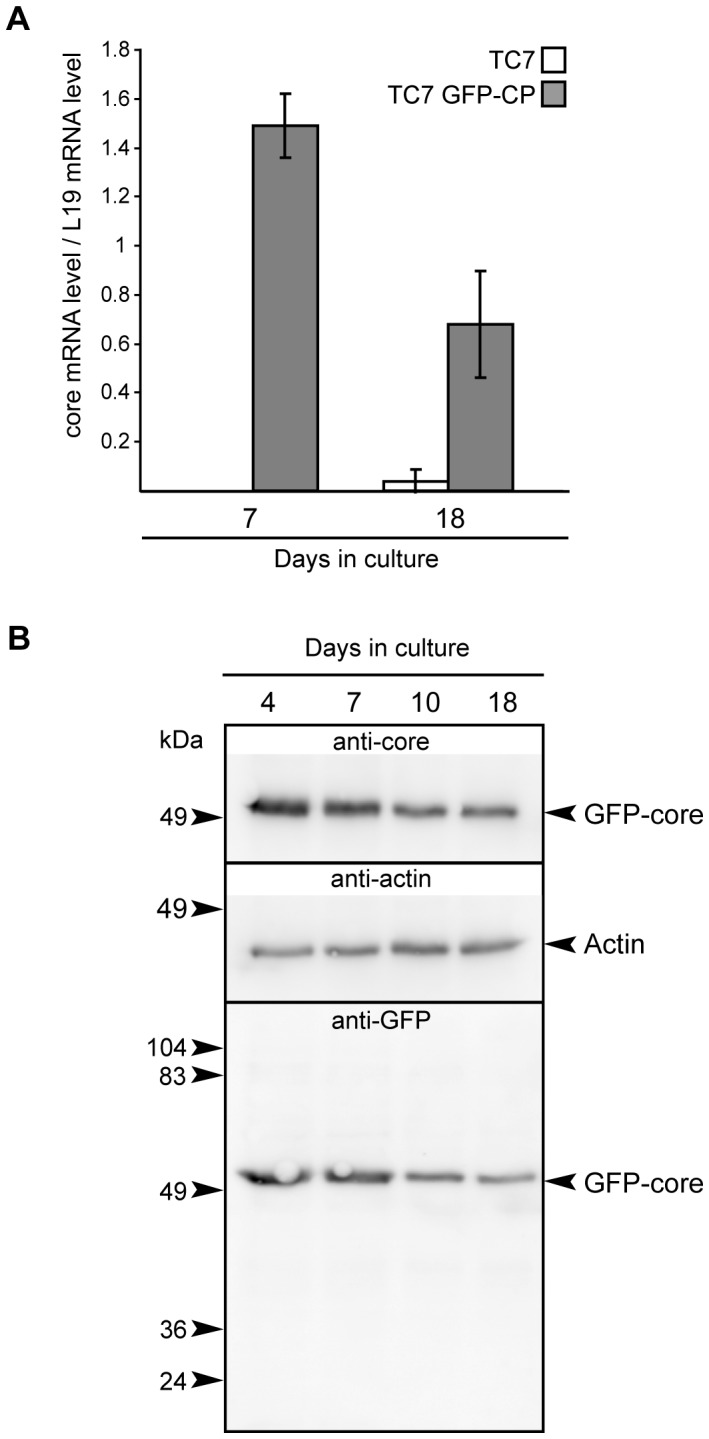
HCV core protein expression in Caco-2/TC7 GFP-CP cells as a function of time in culture. Caco-2/TC7 cells expressing HCV core protein-GFP (TC7 GFP-CP) or not (TC7) were grown on filters for indicated days: confluence is reached on day 7 then cells differentiate i.e. TRL secretion increases gradually with time in culture. Cells were analyzed for expression of HCV core transcripts by quantitative RT-PCR (A) and for HCV core protein and GFP content by western blot (B) using antibodies against HCV core protein or against GFP. Blots were probed for actin as protein loading control.

Caco-2/TC7 GFP-CP cells were cultured on filters and the expression of GFP-CP was analyzed over time at the mRNA and protein levels by quantitative RT-PCR (Fig. 1A) and by western blot (Fig. 1B), respectively. Under these conditions cells reach confluence at day 7 then differentiate into enterocyte-like cells, i.e. gradually acquire their capacity to secrete TRL upon addition of lipid micelles [20]. As shown in Fig. 1A, the core gene was indeed expressed only in Caco-2/TC7 GFP-CP cells and, although the mRNA level of HCV core protein decreased with time, it remained expressed in cells cultured on semi-permeable filters for 18 days, i.e. differentiated Caco-2/TC7 GFP-CP cells, allowing lipid secretion analysis. As shown by western blot, a similar pattern was obtained at the protein level (Fig. 1B). Absence of free GFP was checked (Fig.1B and Fig. S1). No difference in core expression was observed whether lipid micelles were supplied or not (data not shown).

### Impact of HCV Core Protein on Lipid Metabolism in Caco-2/TC7 Cells

We then analyzed whether core protein expression had an impact on the lipid metabolism of Caco-2/TC7 enterocytes.

**Figure 2:**
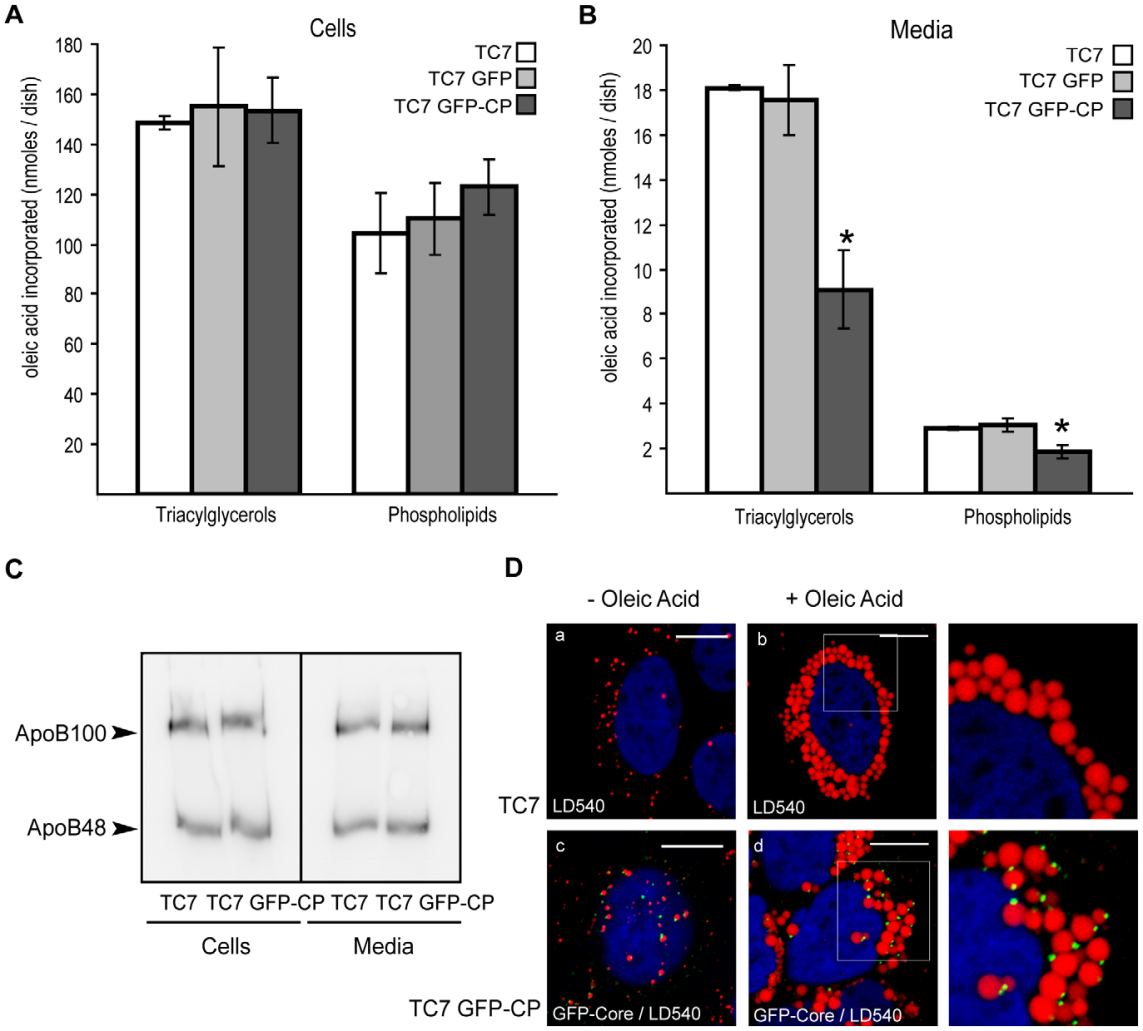
HCV core protein leads to decreased lipid secretion in Caco-2/TC7 enterocytes and localizes on lipid droplets. Caco-2/TC7 cells expressing HCV core protein-GFP (TC7 GFP-CP) and control cells [Caco-2/TC7 cells (TC7) and Caco-2/TC7 cells expressing the empty vector (TC7 GFP)] were cultured on filters for 17 days for differentiation then supplied with lipid micelles for 24 h. Lipid micelles were supplemented (A, B) or not (C) with [1-^14^C]oleic acid. Lipids extracted from cells (A) and basolateral media (B) were fractionated by thin layer chromatography and the radioactivity in the spots was counted. Results for triacylglycerols and phospholipids are shown as means ± SD, expressed as nmoles of oleic acid incorporated per dish, obtained in three independent experiments performed in triplicate (*, p<0.05 compared to control cells). (C) Cell lysates and basolateral media were analyzed for apoB by western blot. (D) Caco-2/TC7 (TC7) and Caco-2/TC7 GFP-CP (TC7 GFP-CP) cells were cultured for one day then incubated (+) or not (−) with 0.6 mM oleic acid/BSA for 24 h. Lipid droplets were visualized using the neutral lipid stain LD540 (red). The core-GFP fusion protein appears in green. Scale bars, 10 µm.

Differentiated Caco-2/TC7 cells, cells expressing the empty vector (Caco-2/TC7 GFP) and cells expressing the GFP-core fusion protein (Caco-2/TC7 GFP-CP) were incubated with lipid micelles containing [1-^14^C]oleic acid for 24 h. After this incubation period, less than 10% of the oleic acid remained in the apical medium (Fig. S2A). This percentage was already reached after 16 h of incubation suggesting that the fatty acid uptake was completed. Cells and media were analyzed for lipid synthesis and secretion (Fig. 2A and B). Lipids were extracted from cell lysates and media, fractionated by TLC and the radioactivity recovered in the TAG and PL spots was measured. A significant decrease in TAG and PL secretion was observed in Caco-2/TC7 GFP-CP cells, compared to control cells (50% and 35% reduction, respectively) (Fig. 2B). Interestingly, the decreased lipid secretion was not accompanied by decreased apoB secretion (Fig. 2C). Since there is one apoB molecule per TRL, this suggests secretion of a similar number of smaller TRL. Since the time course of fatty acid uptake was similar for Caco-2/TC7 and Caco-2/TC7 GFP-CP cells during the incubation period (Fig. S2B), the significant decreased lipid secretion by Caco-2/TC7 GFP-CP cells was not due to a delayed fatty acid uptake that could have led to a delayed TG secretion. The amount of newly synthesized intracellular TAG and PL was not significantly different between core protein-expressing Caco-2/TC7 cells and control cells (Fig. 2A). However, it must be noticed that the approximate 50% decrease of TG secretion by Caco-2/TC7 GFP-CP cells represent about 10 nmoles (Fig. 2B), a value that was indeed within the error bar of the intracellular lipid content since the percentage of secretion is a minor fraction of the total synthesized lipids (about 10%; compare the scales of the y-axis in Fig. 2A and B).

Next, we analyzed the localisation of HCV core protein in Caco-2/TC7 enterocytes by immunofluorescence. After incubation with oleic acid for 24 h, LD were clearly induced, as visualized by the neutral lipid stain LD540 (Fig. 2D, compare+and – oleic acid). Because free GFP was not detected by western blot in Caco-2/TC7 GFP-CP cells (Fig. 1B and Fig. S1), the GFP fluorescence was due to the GFP-CP fusion protein. The GFP-CP fusion protein localized to LD of Caco-2/TC7 GFP-CP cells (Fig. 2D, panel d).

Overall, we have shown that in Caco-2/TC7 enterocytes HCV core protein localizes to LD and leads to a decreased lipid secretion, as observed previously in hepatocytes [29], [31].

### Differential Proteomics of Lipid Droplet Fractions Isolated from Differentiated Caco-2/TC7 GFP-CP Versus Caco-2/TC7 Cells

To gain insight into how HCV core protein interferes with the protein composition of LD, we performed differential proteomics on the LD fractions isolated from differentiated core-expressing Caco-2/TC7 cells versus native Caco-2/TC7 cells, which allowed both the identification and the relative quantification of proteins between the two samples.

**Figure 3:**
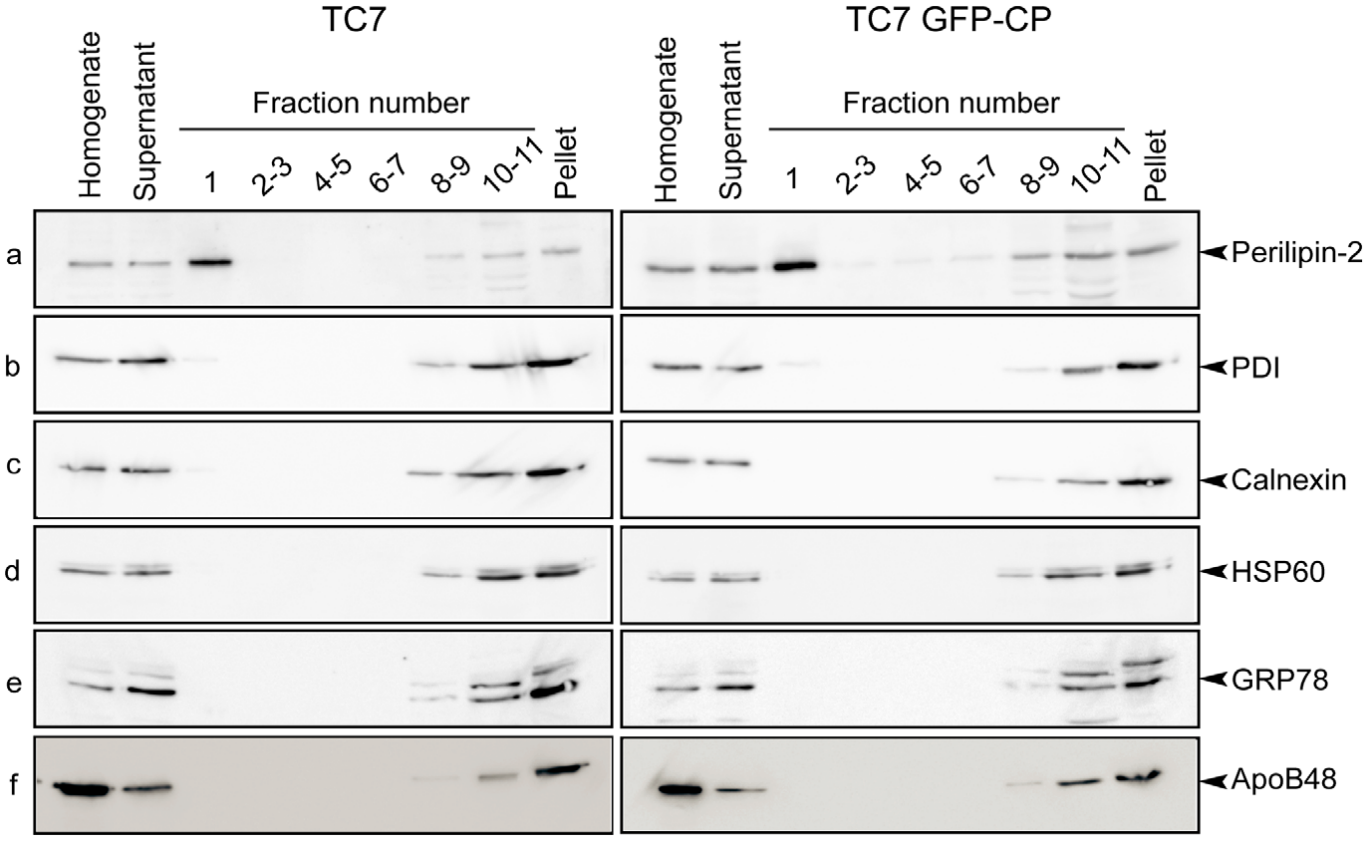
Protein analysis of sucrose gradient fractions prepared from Caco-2/TC7 and Caco-2/TC7 GFP-CP cells. Caco-2/TC7 cells (TC7, left panel) and Caco-2/TC7 GFP-CP cells (TC7 GFP-CP, right panel) were cultured on filters for 17 days then supplied with lipid micelles for 24 h. Cell homogenates were centrifuged for 10 min at 1000×g and the supernatants were fractionated onto sucrose gradients. Top to bottom fractions (1 to 11) and pellets were analyzed by western blot using antibodies specific for (a) perilipin-2/ADRP, (b) protein disulfide isomerase (PDI), (c) calnexin, (d) heat shock protein 60 (HSP 60), (e) 78 kDa glucose-regulated protein (GRP78) and (f) apolipoprotein B48 (ApoB48). The same percentage of each fraction of the sucrose gradient was loaded on gels except for fraction 1 which was 2-fold loaded in order to evaluate the presence of organelle markers with greater sensitivity.

Caco-2/TC7 GFP-CP and Caco-2/TC7 cells were grown on filters for 17 days for differentiation then incubated with lipid micelles for the last 24 h. LD were then isolated using sucrose gradients. While the silver stained gels of the isolated LD fractions were obviously different from the starting cell lysates, the protein profiles of the LD fractions isolated from Caco-2/TC7 and Caco-2/TC7 GFP-CP cells were rather similar (Fig. S3). However, on a 1D-silver stained gel, one single band may contain many proteins and the quantitative modification of one of them may not be observed, in particular if its relative amount is low. As described previously for Caco-2/TC7 cells [10], the lowest density fraction (fraction number 1) isolated from Caco-2/TC7 GFP-CP was highly enriched in perilipin-2/ADRP, a marker of lipid droplets (Fig. 3a). Because differentiated Caco-2/TC7 cells secrete TRL, which might co-purify with cytosolic LD, the sucrose gradient fractions were examined for the presence of apoB48, the non-exchangeable apolipoprotein present in TRL. Reported results in Fig. 3f indicate clearly that fraction 1 was not contaminated by TRL. Indeed, apoB48 was detected in the bottom fractions, which contained membranes (including microsomes). Fractions were also tested for PDI (protein disulfide isomerase), calnexin and GRP78 (78 kDa glucose-regulated protein), all being microsomal proteins. These proteins, routinely identified by proteomics in LD fractions (for review, see [9]), could hardly be detected by western blot in LD-containing fractions (Fig. 3b, c, e). Finally, the mitochondrial marker HSP60 could not be detected in the LD fraction (Fig. 3d).

For differential proteomics, proteins contained in the LD fractions isolated from Caco-2/TC7 and Caco-2/TC7 GFP-CP cells were digested with trypsin then labeled with different iTRAQ reagents. Peptides were identified by LC-MS/MS. This differential proteomic approach allowed the relative quantitative identification of 42 different proteins (Table 2). An iTRAQ ratio greater than one indicated a higher abundance of this protein in LD isolated from core-expressing Caco-2/TC7 cells than control Caco-2/TC7 cells. Conversely, a ratio less than one indicated that this protein was less abundant in the LD fraction from Caco-2/TC7 GFP-CP cells than Caco-2TC7 cells. The range of the ratios was rather limited (i.e. 0.389–1.837) and consistent with the similarity of the protein profiles observed on 1D-silver stained gels of LD fractions isolated from Caco-2/TC7 or Caco−/TC7 GFP-CP cells.

The most abundant protein identified was perilipin-2 (data not shown), as reported previously for Caco-2/TC7 cells [10] and confirming the validity of this approach. Remarkably, 21 proteins (50%) were directly related to lipid metabolism, including LD coat proteins (perilipins), enzymes involved in fatty acid activation or in the synthesis or degradation of acylglycerols, phospholipids, cholesterol and steroids (Table 2). Proteins involved in intracellular trafficking (Rabs) or known to be associated with ER were also identified.

The two proteins that were most up-regulated in LD fractions from Caco-2/TC7 GFP-CP cells were both involved in steroid metabolism. 17β-hydroxysteroid dehydrogenase type 2 (DHB2) is a member of the SDR (short chain dehydrogenase/reductase) superfamily [43] and catalyzes the oxidative conversion between 17-ketosteroid and 17β-hydroxysteroid pairs like estrone and estradiol or androstenedione and testosterone [41]. 3β-hydroxysteroid dehydrogenase (3BHS1) is responsible for the oxidation and isomerization of Δ5-3β-hydoxysteroid precursors to form Δ4-ketosteroids and plays a crucial role in the biosynthesis of all classes of hormonal steroids. However, because of its variability, the iTRAQ ratio obtained for 3BHS1 did not reach statistical significance. Other up-regulated proteins were monoglyceride lipase (MGLL), which hydrolyses monoacylglycerides to free fatty acids and glycerol, the PLIN protein perilipin-2 and lysophosphatidylcholine acyltransferase 2 (PCAT2), which reacylates lysophosphatidylcholine into phosphatidylcholine.

In contrast, microsomal triglyceride transfer protein (MTP) and its subunit protein disulfide isomerase A1 (PDIA1), which are required for TRL assembly and lipid droplet production in the ER lumen, were strongly decreased in LD-containing fractions isolated from Caco-2/TC7 GFP-CP cells, as were a number of ER-associated chaperones. Additionally, unlike perilipin-2, perilipin-3 was decreased.

### Protein Expression and Localisation of Selected Proteins in Caco-2/TC7 and Caco-2/TC7 GFP-CP Cells

**Figure 4:**
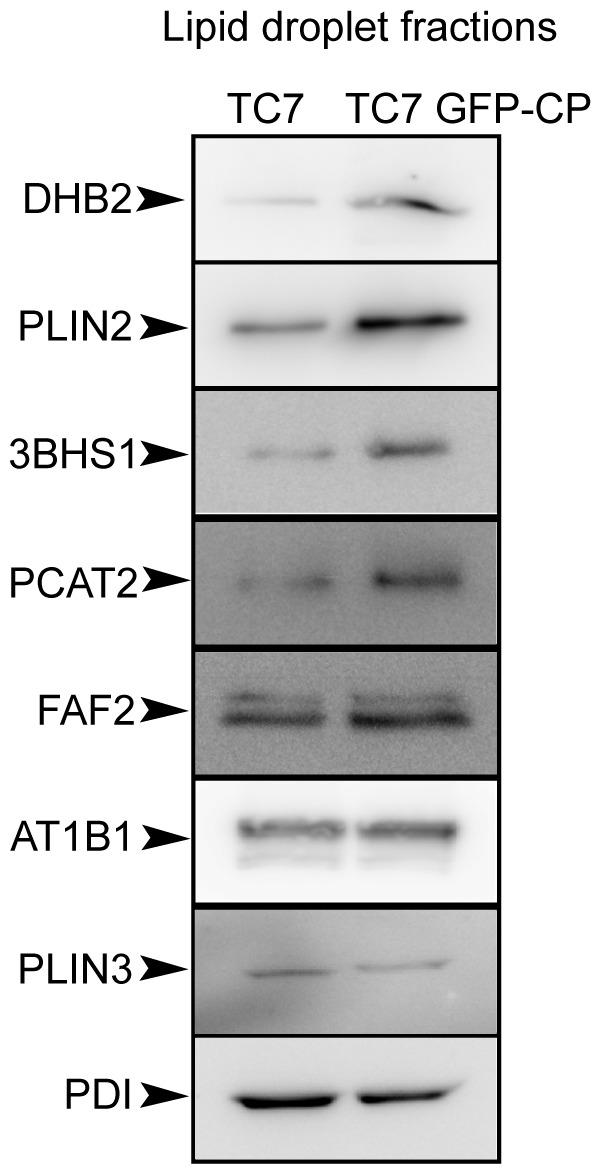
Western blot analysis of some proteins identified by the differential proteomic approach in the lipid droplet fractions isolated from Caco-2/TC7 (TC7) and Caco-2/TC7 GFP-CP (TC7 GFP-CP) cells. The lipid droplet fractions (fraction 1) were prepared as described in figure 3, freeze-dried for concentration and analyzed by western blot using antibodies against 17β-hydroxysteroid dehydrogenase type 2 (DHB2), perilipin-2/ADRP (PLIN2), 3-beta-hydroxysteroid dehydrogenase (3BHS1), lysophosphatidylcholine acyltransferase 2 (PCAT2), FAS-associated factor 2/UBXD8 (FAF2), Na+/K+ATPase α1 (AT1B1), perilipin-3/TIP47 (PLIN3) and protein disulfide isomerase (PDI). The amount of material loaded per well was 10 times higher than in Fig.3, fraction 1.

Results obtained by this quantitative proteomic approach were confirmed by immunoblotting of LD fractions isolated from differentiated Caco-2/TC7 and Caco-2/TC7 GFP-CP cells supplied with lipid micelles for 24 h (Fig. 4). Because there is no suitable loading control for LD fractions and since there was no modification of the TAG content between the cell lines (data not shown), experiments were performed by starting with equal numbers of cells and loading equal volumes. To improve the immunodetection of the proteins, LD fractions isolated from Caco-2/TC7 or Caco-2/TC7 GFP-CP cells were freeze-dried in order to load ten times more material per well than for western blots shown on Fig. 3. The Western blots performed using available antibodies against eight proteins identified in the proteomic study (Table 2) confirmed the relative increase, stability or decrease of these proteins between LD fractions isolated from Caco-2/TC7 cells expressing core protein or not (Fig. 4). As a consequence of the higher amount of material loaded on the gels, PDI, which was detected hardly when using unconcentrated isolated LD fractions (Fig. 3), became detectable.

**Figure 5:**
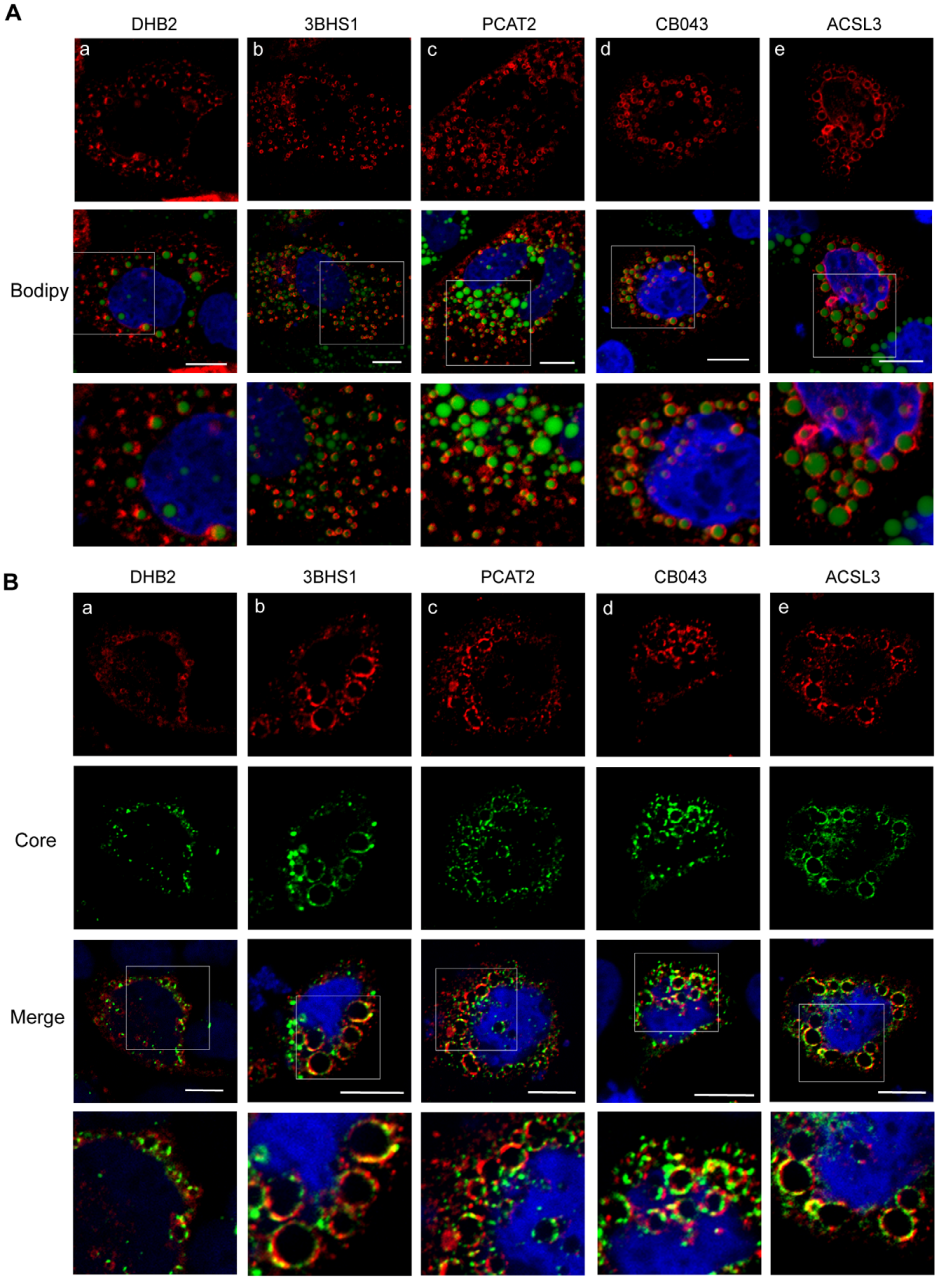
Proteins identified by LC-MS/MS in the lipid droplet fractions isolated from Caco-2/TC7 cells localise to lipid droplets (A) and co-localise with HCV core protein around lipid droplets (B). Caco-2/TC7 cells were transfected with plasmids encoding the proteins of interest fused to a myc-tag (A) or double transfected with plasmids expressing proteins of interest fused to a myc-tag and the core expressing plasmid pGFP-CP (B), and incubated with 0.6 mM oleic acid/BSA for 24 h to induce lipid droplet formation. The myc-tag was detected with mAb9E10 and Alexa Fluor 568–conjugated anti-mouse IgG (red) and the core protein by GFP fluorescence (green). (a) 17β-hydroxysteroid dehydrogenase type 2 (DHB2), (b) 3-beta-hydroxysteroid dehydrogenase (3BHS1), (c) lysophosphatidylcholine acyltransferase type 2 (PCAT2), (d) UPF0554 C2orf43 (CB043) and (e) long-chain-fatty-acid–CoA ligase 3 (ACSL3). Scale bars, 10 µm.

Next, we analyzed by confocal microscopy the intracellular localisation of some of the identified proteins that were connected to lipid metabolism. We selected DHB2, 3BHS1 and PCAT2, which were found up-regulated in Caco-2/TC7 GFP-CP cells compared to Caco-2/TC7 cells, and ACSL3 and CB043, which were not altered but are frequently found in proteomic studies [9]. Caco-2/TC7 cells were transfected with plasmids encoding the proteins of interest fused to a N-terminal myc-tag, then LD formation was induced by incubation with 0.6 mM oleic acid for 24 h. The proteins were visualised using specific anti-myc antibody and LD were visualised by Bodipy 493/503. As shown in Fig. 5A, a clear LD-associated localisation was detected for all the tested proteins. However, whereas the localisation of 3BHS1 and CB043 was almost exclusively around LD, the localisation of DHB2, PCAT2 and ASCL3 around LD was partial. Double transfections of Caco-2/TC7 cells with pGFP-CP, expressing GFP fused to the HCV core gene serotype 1b, and the proteins listed above showed co-localisation of the studied proteins with the core protein around lipid droplets (Fig. 5B).

### mRNA Levels of Selected Proteins in Caco-2/TC7 Cells, Caco-2/TC7 GFP-CP Cells and Human Small Intestine

**Figure 6:**
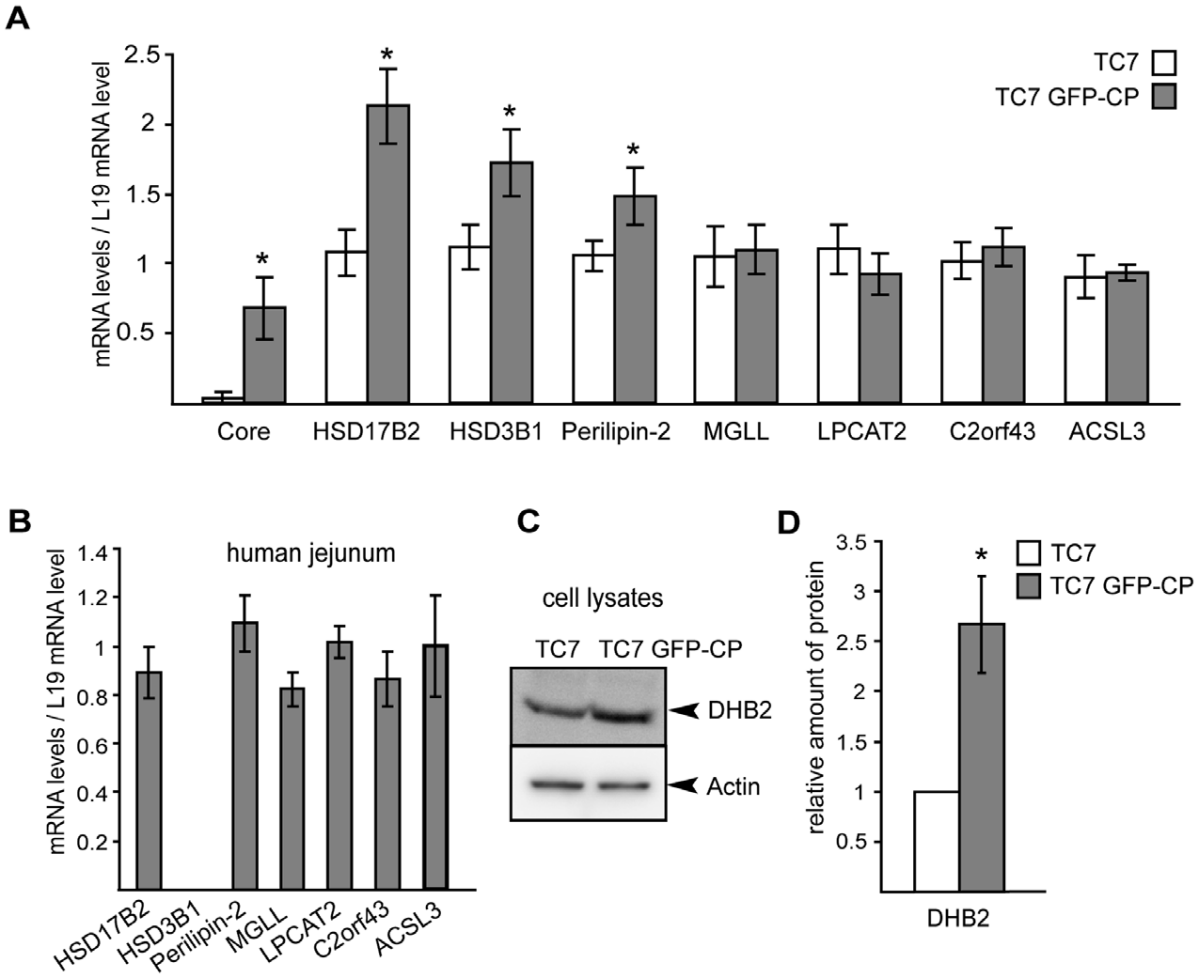
Gene and protein expression in Caco-2/TC7 cells (TC7), Caco-2/TC7 GFP-CP cells (TC7 GFP-CP) and human jejunum of some proteins identified by LC-MS/MS in lipid droplet fractions. (A) Caco-2/TC7 and Caco-2/TC7 GFP-CP cells were cultured on filters for 17 days then supplied with lipid micelles for 24 h. mRNA levels were measured by quantitative RT-PCR for core (HCV core protein), HSD17B2 (17β-hydroxysteroid dehydrogenase type 2), HSD3B1 (3-beta-hydroxysteroid dehydrogenase), PLIN2 (perilipin-2), MGLL (monoacylglycerol lipase), LPCAT2 (lysophosphatidylcholine acyltransferase 2), C2orf43 (UPF0554 protein C2orf43) and ACSL3 (long-chain-fatty-acid–CoA ligase 3). (B) mRNA levels for the same genes were quantified in human jejunum mRNA samples. (C) Lysates of Caco-2/TC7 and Caco-2/TC7 GFP-CP cells were analyzed by western blot for 17β-hydroxysteroid dehydrogenase type 2 (DHB2) and actin. (D) The immunoblot shown in C was quantified and standardized to actin used as the loading control. Results shown are the means ± SD from three independent experiments performed in triplicate, except for human jejunum (one sample measured in triplicate). *, p<0.05 compared to control cells.Caco-2 cell line derives from a human epithelial colorectal adenocarcinoma and TC7 is a clone of Caco-2 cells [37]. Although these cells differentiate such that their phenotype resembles absorptive enterocytes of the small intestine, it still remains a cell line with a cancerous origin and thus proteins might be differently expressed in normal cells from human intestine. Therefore, to assess the physiological relevance of these results, we performed similar experiments on mRNA isolated from human small intestine (Fig. 6B). All above-mentioned genes were expressed in human small intestine except HSD3B1. Therefore, since 3BHS1 protein was also not significantly up-regulated in LD isolated from Caco-2/TC7 GFP-CP as compared to Caco-2/TC7 cells, it was not studied further.

Since DHB2, 3BHS1, MGLL, perilipin-2 and PCAT2 were up-regulated in LD fractions of Caco-2/TC7 GFP-CP cells as compared to Caco-2/TC7 cells (Table 2), we analyzed whether these proteins were up-regulated at the mRNA level as well. As shown in Fig. 6A, the mRNA levels of HSD17B2, HSD3B1, perilipin-2 and indeed, of HCV core protein, were significantly higher in Caco-2/TC7 GFP-CP cells than in Caco-2/TC7 cells. However, the mRNA levels were unchanged for MGLL and LPCAT2, proteins that were found up-regulated in the proteomic approach, as well as for C2orf43 and ACSL3, proteins that were not modified.

The Caco-2 cell line derives from a human epithelial colorectal adenocarcinoma and TC7 is a clone of Caco-2 cells [37]. Although these cells differentiate such that their phenotype resembles absorptive enterocytes of the small intestine, it still remains a cell line with a cancerous origin and thus proteins might be differently expressed in normal cells from human intestine. Therefore, to assess the physiological relevance of these results, we performed similar experiments on mRNA isolated from human small intestine (Fig. 6B). All above-mentioned genes were expressed in human small intestine except HSD3B1. Therefore, since 3BHS1 protein was also not significantly up-regulated in LD isolated from Caco-2/TC7 GFP-CP as compared to Caco-2/TC7 cells, it was not studied further.

We focused on the protein DHB2 that, with an iTRAQ ratio of 1.837±0.092, was the most up-regulated LD-associated protein in Caco-2/TC7 GFP-CP cells as compared to Caco-2/TC7 cells. DHB2 is expressed in the gastrointestinal tract as well as in the Caco-2 cell line [44], [45], and Fig. 6A and B). To distinguish a local LD enrichment from an overall higher cellular amount, we analyzed DHB2 levels in cell lysates by western blot. Fig. 6C and D show that DHB2 was significantly overexpressed in Caco-2/TC7 GFP-CP cell lysates compared to Caco-2/TC7 cells.

Overall, our results indicate that DHB2 localizes partially to LD and that HCV core protein expression leads to an increased expression of DHB2 both at the mRNA and protein levels.

### DHB2 Activity in Caco-2/TC7 and Caco-2/TC7 GFP-CP Cells

**Figure 7:**
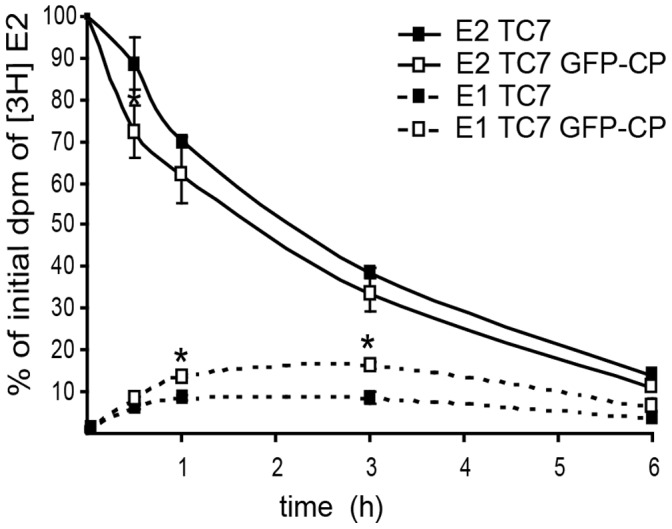
DHB2 activity in Caco-2/TC7 (TC7) and Caco-2/TC7 GFP-CP (TC7 GFP-CP) cells. Cells were cultured on filters for 17 days then incubated with [^3^H] estradiol (E2) for the indicated times. Steroids were extracted from the basolateral media and separated by TLC. The radioactivity in the spots corresponding to estradiol (E2) and estrone (E1) was quantified by scintillation counting. Results are expressed as percentage of dpm of [^3^H]estradiol present in medium at time 0. Data are mean ± SD from triplicate determinations of three independent experiments. *, p<0.05 compared to control cells.

To determine whether DHB2 is active in Caco-2/TC7 cells, DHB2 activity was measured using estradiol (E2) as a substrate. The metabolism of E2 is complex as E2 is converted by DHB2 into estrone (E1) but other E2 metabolites are also produced, including glucuronide derivatives and methyl esters [46]. Differentiated cells were incubated in medium containing [^3^H] E2 and quantification of [^3^H] E1 in basolateral medium was measured as a function of time. As expected, Caco-2/TC7 GFP-CP cells, which expressed more DHB2 than Caco-2/TC7 cells, converted [^3^H] E2 into [^3^H] E1 more rapidly (Fig. 7).

### Impact of DHB2 Depletion on the Lipid Metabolism of Caco-2/TC7 and Caco-2/TC7 GFP-CP Cells

**Figure 8:**
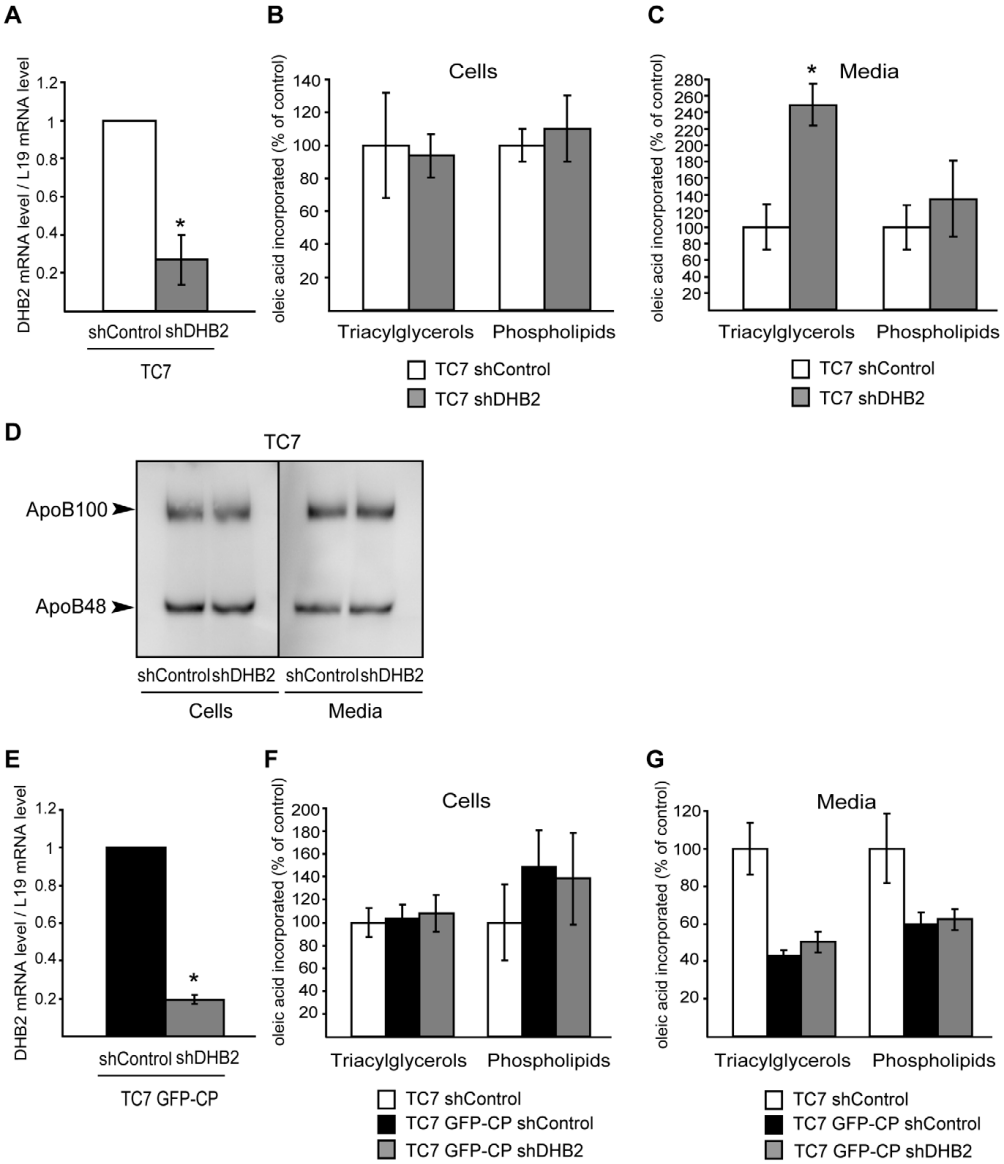
Depletion of DHB2 leads to increased lipid secretion by Caco-2/TC7 cells. Caco-2/TC7 cells (A−D) and Caco-2/TC7 GFP-CP cells (E−G) cultured on filters were transduced with lentiviral vectors expressing shRNA directed against the luciferase gene (shControl) or HSD17B2 (shDHB2). After 17 days in culture, cells were incubated with lipid micelles for 24 h. Lipid micelles were supplemented (B, C, F, G) or not (A, D, E) with [1-^14^C]oleic acid. The efficiency of silencing in Caco-2/TC7 cells (A) and Caco-2/TC7 GFP-CP cells (E) was analyzed by quantitative RT-PCR. shControl values were set at 1. Lipids extracted from cells (B and F) and basolateral media (C and G) were fractionated by thin layer chromatography and the radioactivity in triacylglycerols and phospholipids was counted. Results are expressed as a percentage of control cells (TC7 shControl). Data are means ± SD of at least four independent experiments. *, p<0.05 compared to control cells.

If the decreased lipid secretion observed in Caco-2/TC7 GFP-CP cells compared to Caco-2/TC7 cells was related to the increased DHB2 expression, silencing DHB2 by shRNA should lead to increased lipid secretion. To test this hypothesis, Caco-2/TC7 transduced with a lentiviral vector expressing shDHB2 or shControl were cultured for 17 days for differentiation then incubated with micelles containing [1-^14^C]oleic acid for 24 h. Lipids extracted from cells and media were separated by TLC and the radioactivity in the resulting spots was measured. The efficiency of silencing was checked by quantitative RT-PCR (Fig. 8A). While TAG and PL synthesis was not altered in cells depleted for DHB2 compared to control cells (Fig. 8B), TAG secretion was increased 2.5 times (Fig. 8C). The rise in TAG secretion was not accompanied by a modification of apoB secretion (Fig. 8D), suggesting the secretion of larger TRL by cells depleted for DHB2 compared to control cells.

Similar experiments were performed on Caco-2/TC7 GFP-CP cells (Fig. 8E–G). Although not statistically significant, DHB2 depletion resulted in increased TAG secretion (17% increase in Caco-2/TC7 GFP-CP shDHB2 cells as compared to TC7 GFP-CP shControl cells).

### Effect of Estradiol and Testosterone on Lipid Metabolism in Caco-2/TC7 Cells

**Figure 9:**
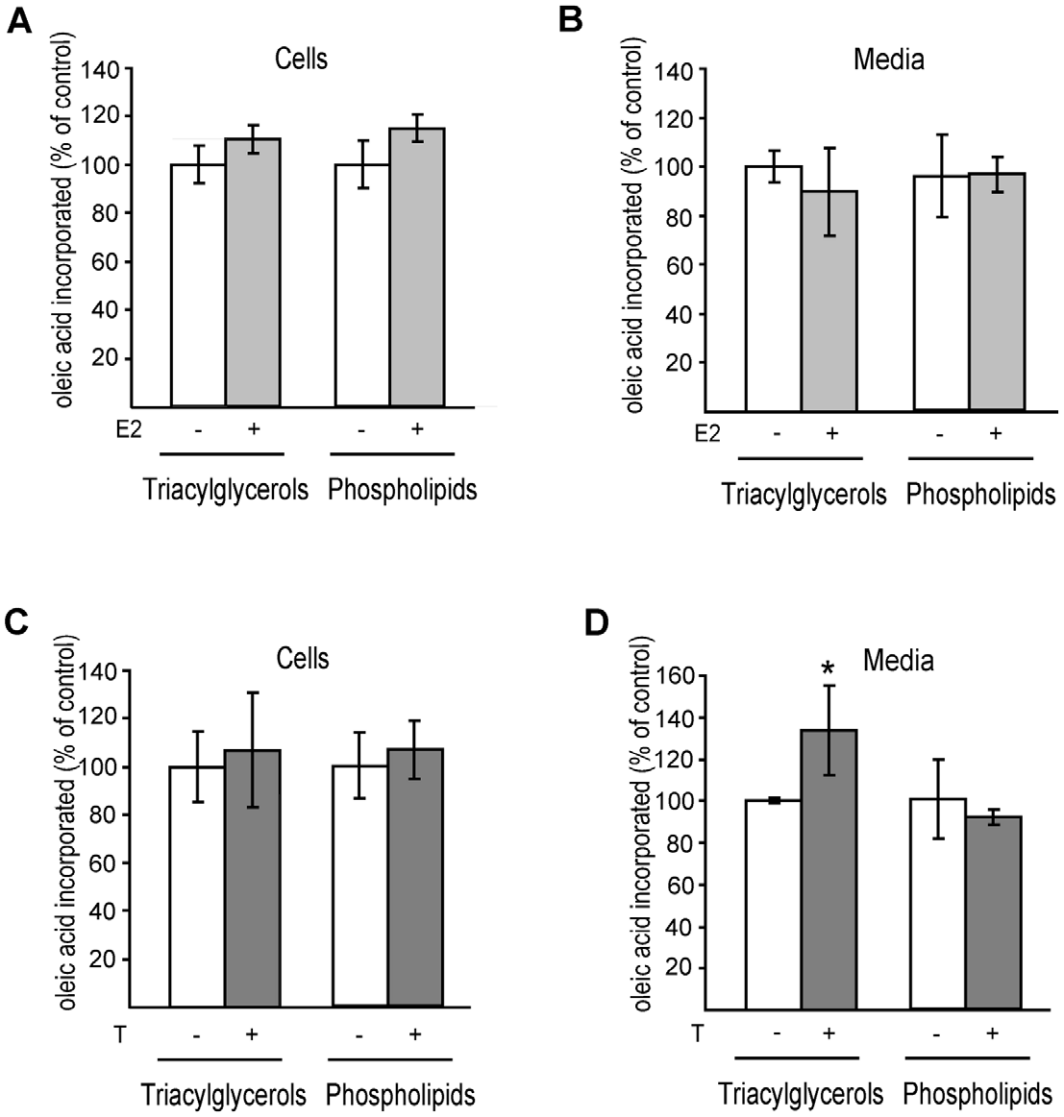
Effect of estradiol (E2) and testosterone (T) on lipid synthesis and secretion by Caco-2/TC7 cells. Caco-2/TC7 cells were cultured on filters for 17 days then incubated with lipid micelles supplemented with [1-^14^C]oleic acid, in the presence or not of estradiol (A, B) or testosterone (C, D) for 24 h. Lipids extracted from cells (A and C) and basolateral media (B and D) were fractionated by thin layer chromatography and the radioactivity in triacylglycerols and phospholipids was counted. Results are expressed as percentage of control cells. Data are means ± SD of four independent experiments. *, p<0.05 compared to control cells.

Since DHB2 catalyses the conversion of estradiol (E2) and testosterone (T) into biologically inactive forms, estrone and androstenedione, respectively, we analysed whether E2 and/or T had an effect on lipid secretion by Caco-2/TC7 cells (Fig. 9). Caco-2/TC7 cells cultured for 17 days for differentiation were incubated with micelles containing [1-^14^C]oleic acid and supplemented with E2 or T for 24 h. Lipids extracted from cells and media were separated by TLC and the radioactivity in the resulting spots was measured. Neither estradiol nor testosterone had an impact on lipid synthesis (Fig. 9A and C, respectively), compared to control cells. However, while estradiol had no effect on lipid secretion (Fig. 9B), testosterone induced an increased TAG secretion (Fig. 9D).

Overall, results suggest that, in Caco-2/TC7 cells, DHB2 can modulate lipid secretion through its capacity to inactivate testoterone.

## Discussion

Our objective in this study was to identify LD-associated proteins that could be involved in the balance between TAG storage and secretion in enterocytes. For this, we took advantage of the ability of HCV core protein that localizes to LD to modify this balance since, in hepatocytes, HCV core protein impairs TRL secretion and induces lipid accumulation in the cytosol [29], [30], [31], [47]. To obtain an overview of the proteins that were quantitatively modified on LD isolated from cells expressing HCV core protein or not, we used a differential proteomic approach: LC-MS/MS of iTRAQ-labeled peptides obtained by trypsin digested proteins. As model of enterocytes, we used Caco-2/TC7 cells, which are the only cell culture model of human enterocytes able to secrete TRL and store TAG as cytosolic LD that can be later mobilized for TRL production [20].

In Caco-2/TC7 enterocytes, the expression of HCV core protein led to a 50% decrease in TAG secretion. Since the amount of apoB secreted was unaltered, this suggests the secretion of a similar number of smaller LRT and therefore the impaired production of LD in the ER lumen. Also, these results show that the effect of HCV core protein on the lipid metabolism is observed both in hepatocytes and enterocytes, suggesting common mechanisms for this effect.

Using iTRAQ quantitative proteomics, we identified and differentially quantified a total of 42 proteins in the LD fractions isolated from Caco-2/TC7 GFP-CP cells, compared to those from Caco-2/TC7 cells. Interestingly, 50% of these proteins were related to lipid metabolism, including the LD coat proteins perilipins and enzymes involved in fatty acid activation or acylglycerol, phospholipid, cholesterol and steroid synthesis or degradation. Among them, DHB2 and ACSL5 (long-chain-fatty-acid-CoA ligase 5), which belong to the HSD (hydroxysteroid dehydrogenase) and ACSL families, respectively, were identified for the first time in LD fractions. It has been shown that other members of these families are present in LD fractions [9], [48], and in hepatic cells ACSL3 knockdown was shown to result in decreased apoB secretion [49]. However, proteomics showed no quantitative modification of ACSL3 or ACSL5 suggesting that they are not involved in the effect of HCV core protein on lipid secretion in enterocytes. ACSL5 was not identified previously on LD most probably because its expression is particularly high in epithelial cells of the small intestine as compared to other organs [50], [51]. It is noteworthy that eight proteins were linked to sterol metabolism: DHB2, 3BHS1, NB5R3, DHB11, DHB7, NSDHL, DHRS3 and ERG1. While enzymes involved in cholesterol biosynthesis have been routinely identified in proteomic studies [9], [10], [52], the identification of enzymes involved in steroid metabolism was more surprising because the intestine is not reputed as a steroidogenic organ. However, as suggested for DHB11 [53], these enzymes may be involved in the metabolism of diet-derived or oxidized hydrophobic, potentially toxic molecules. Though liver is reputed to be the major xenobiotic-metabolizing organ, enterocytes are in contact with a large variety of xenobiotics and intestine contributes to the first steps of detoxification [54], [55].

By confocal microscopy of cells expressing these proteins of interest, we confirmed the localization around LD of CB043, which was routinely identified in LD fractions, but only by proteomics [9], [10]. CB043 has no function assigned yet but contains homologies for an esterase-lipase superfamily domain and an abhydrolase-6 domain. Our proteomic analysis showed no quantitative modification of CB043 suggesting that the CB043 protein amount on LD per se is not involved in the effect of HCV core protein on lipid secretion in these cells. Indeed, next to the protein amount, the protein/enzyme activity of CB043 may be controlled by many factors including post-translational modifications, cofactors or substrate availability.

Six Rab proteins that are involved in vesicular traffic and are routinely identified in LD fractions [9], [56] were identified, but none of them were significantly differentially expressed between the two cell lines.

Concerning the perilipin family that contributes to the protein coat of LD, perilipin-2 was significantly enriched while perilipin-3 was depleted in the LD fractions isolated from Caco-2/TC7 GFP-CP, compared to Caco-2/TC7 cells. Perilipin-2 and perilipin-3 are the only PLIN family proteins expressed in enterocytes [18]. In adipocytes, it has been shown that the earliest deposits of neutral lipids are coated with perilipin-3 [57] and, more recently, that perilipin-3 is involved in the biogenesis of LD [58]. In enterocytes, Lee et al. [59] suggested that perilipin-3 plays a role in the synthesis of LD from newly synthesized TAG, while perilipin-2 plays a role in the stabilization of TAG stored in longer term. Moreover, overexpression of perilipin-2 reduces lipolysis catalyzed by Adipose Triglyceride Lipase (ATGL) [60], which was identified in LD fractions of Caco-2/TC7 cells [10]. Overall, the modification of the perilipin-2/perilipin-3 balance induced by HCV core protein in Caco-2/TC7 cells favours stabilisation of the LD. This is in good agreement with the recent report describing that LD-localized core protein slows down the turnover of TAG in LD [61]. However, there was no modification of the TAG content between the cell lines. To go further, it would be worth studying other key enzymes involved in the TAG hydrolysis/reesterification process such as lipases and acyltransferases as well as examining TAG turnover by using pharmacological inhibitors.

Interestingly, a number of chaperones associated with the ER, such as PDI isoenzymes, and proteins involved in lipoprotein secretion, such as MTP, were strongly depleted in LD-associated fractions isolated from Caco-2/TC7 GFP-CP cells, compared to Caco-2/TC7 cells. Although the identification by proteomics, in LD fractions, of proteins reputed to have an ER location has been often considered as a contamination, it is now accepted that there are strong physical relationships between ER and LD [15], [16]. The iTRAQ quantitative approach clearly indicated a decreased association of LD with ER in cells expressing HCV core protein. In core protein expressing hepatocytes, it has been shown previously that the MTP amount is not modified but MTP activity is impaired, a mechanism suggested to contribute to the development of steatosis and the decreased secretion of TRL by these cells [29]. An additional, non exclusive, hypothesis is that core expression leads to a decreased association of LD to ER membranes, which may contribute to the impaired secretion of lipids observed in core-expressing Caco-2/TC7 cells versus control cells.

Additionally to perilipin-2, other proteins were found enriched in the LD fractions of Caco-2/TC7 cells expressing HCV core protein versus control cells. Immunofluorescence studies confirmed the LD-associated localization of DHB2, 3BHS1 and PCAT2. Moreover, stable HCV core expression in Caco-2/TC7 cells led to altered gene expression for some of these proteins. DHB2 was the most enriched around LD, up-regulated at the mRNA and protein levels, and has never been described associated to LD or connected to HCV core protein. DHB2 belongs to the family of 17ß-hydroxysteroid dehydrogenases that occupy pivotal positions in steroid metabolism pathways, regulating the intracellular concentrations of active (E2 and T) and inactive (E1 and androstenedione) steroid pairs. DHB2 inactivates E2 and T into E1 and androstenedione, respectively, while the reverse reaction is catalysed by DHB1 for E1 and DHB3 for androstenedione [41]. In Caco-2 cells, these isoforms are present [44]. Indeed, DHB2 is expressed in the human gastrointestinal tract, particularly in the epithelial cells of the small intestine [45]. In these cells, DHB2 has been suggested to be involved in the inactivation of endogenous and exogenous active sex steroids. DHB2 also has a 20α-HSD activity [62]. Possibly, DHB2 is an enzyme with unknown yet substrates and thus with additional functions [63].

Using estradiol as a substrate for DHB2, we showed that Caco-2/TC7 GFP-CP cells, which overexpressed DHB2 compared to control cells, were more potent at transforming estradiol into estrone, as expected. The silencing of DHB2 in Caco-2/TC7 cells caused an increase of TAG secretion, confirming the capacity of DHB2 to impair TAG secretion by Caco-2/TC7 cells. Similar experiments performed on Caco-2/TC7 GFP-CP cells showed only a partial, not significant, restoration of TAG secretion. Keeping in mind that HCV core protein led to the quantitative modification of many proteins in LDs fractions, the lack of a clear-cut effect by modulating the amount of one single protein was not that surprising. The effect of DHB2 on TAG secretion could be mediated by its enzyme activity on substrates, or by coating the LD surface and impairing the access of other proteins. We thus tested the effect of E2 and T on lipid secretion by Caco-2/TC7 cells and found that, while E2 had no effect, T led to an increase of TAG secretion. In humans, sex steroids are known to exert profound and complex effects including on lipid metabolism [64], [65], [66]. However, data from literature on the effect of T on serum lipid levels are contradictory [67], [68]. The effects of steroid hormones are mediated through interaction with specific intracellular receptors, which are also present in the gastrointestinal tract [69]. To our knowledge, no data describing the impact of T on lipid secretion by intestine are available. We show for the first time that T leads to an increased lipid secretion in enterocyte-like Caco-2/TC7 cells, although the effect remains modest. Gathering all the present results, we can formulate the following model: DHB2, which is upregulated in HCV core-expressing Caco-2/TC7 cells, leads to a more rapid inactivation of steroid hormones, including testosterone, that stimulates lipid secretion in Caco-2/TC7 enterocytes.

In summary, by a differential proteomic approach, we identified proteins on lipid droplets that are altered by HCV core protein in Caco-2/TC7 enterocytes. Because HCV core protein led to a decreased TRL secretion, the identified players are potentially involved in the control of the balance between lipid storage, as LD, and secretion, as TRL, in Caco-2/TC7 enterocytes. Factors modifying this balance may be simple proteins, as shown for DHB2, or the ratio between two proteins, such as the perilipin-2/perilipin-3 ratio, or the extent of LD association to organelles, such as the ER. Further studies on these identified factors will help to gain more knowledge about this white spot on the map of cellular pathways i.e. the crosstalk of cytosolic LD and TRL formation in the ER. High levels of intestinally derived lipoproteins are associated with increased cardiovascular risk and there is evidence of altered TRL secretion by intestine in pathological conditions, such as insulin resistance, type II diabetes and obesity [22], [23], [24], [25]. This altered TRL secretion may result from an imbalance between the cytosolic and luminal LD dynamics and the underlying mechanisms are important to characterize. Additionally, since we used HCV core protein to perturb the TAG balance, our results may help to further characterize the effect of HCV core protein on LD metabolism, which is necessary for HCV replication and production in hepatocytes.

## Supporting Information

Figure S1 **Western blot analysis of GFP-HCV core protein produced by the stably transfected Caco-2/TC7 GFP-CP cell line.** Caco-2/TC7 cells expressing GFP-HCV core protein (TC7 GFP-CP) were grown on filters for 18 days then cell lysates were analyzed for GFP (A) or HCV core protein (B). Cell lysates from Caco-2/TC7 cells, Caco-2/TC7 cells transiently transfected with plasmids encoding GFP or HCV core protein are included as controls.
(TIF)

Figure S2 **Time course of oleic acid incorporation into Caco-2/TC7 (TC7) and Caco-2/TC7 GFP-CP (TC7 GFP-CP) cells.** Cells were grown on filters for 17 days then incubated for various durations with lipid micelles supplemented with [1-^14^C]oleic acid. The radioactivity remaining in the apical medium was counted and expressed as percentage of the radioactivity present at time 0 (A). Radioactivity contained in cell lysates was counted and expressed as nmoles of oleic acid incorporated per dish (B). Data are means ± SD of three independent experiments performed in duplicate.
(TIF)

Figure S3 **Silver stained gels of lipid droplet fractions and cell lysates from Caco-2/TC7 cells (TC7) and Caco-2/TC7 GFP-CP (TC7 GFP-CP) cells.** Cells were cultured on filters for 17 days then supplied with lipid micelles for 24 h. Lipid droplet fractions were prepared as described in the Materials and Methods section, freeze-dried for concentration and one tenth of the lipid droplet fraction was loaded per well. One µg of cell lysates was loaded per well. Proteins were separated by 10% SDS-PAGE and silver stained.
(TIF)

Table S1 **Oligonucleotide primers used for gene expression analysis.**
(DOC)
