# Supplementary figures and images for: Proteomic Analysis of Lipid Droplets from Caco-2/TC7 Enterocytes Identifies Novel Modulators of Lipid Secretion
Source: PLoS One. 2013 Jan 2;8(1):e53017. doi: 10.1371/journal.pone.0053017 (PMC3534623; doi:10.1371/journal.pone.0053017)

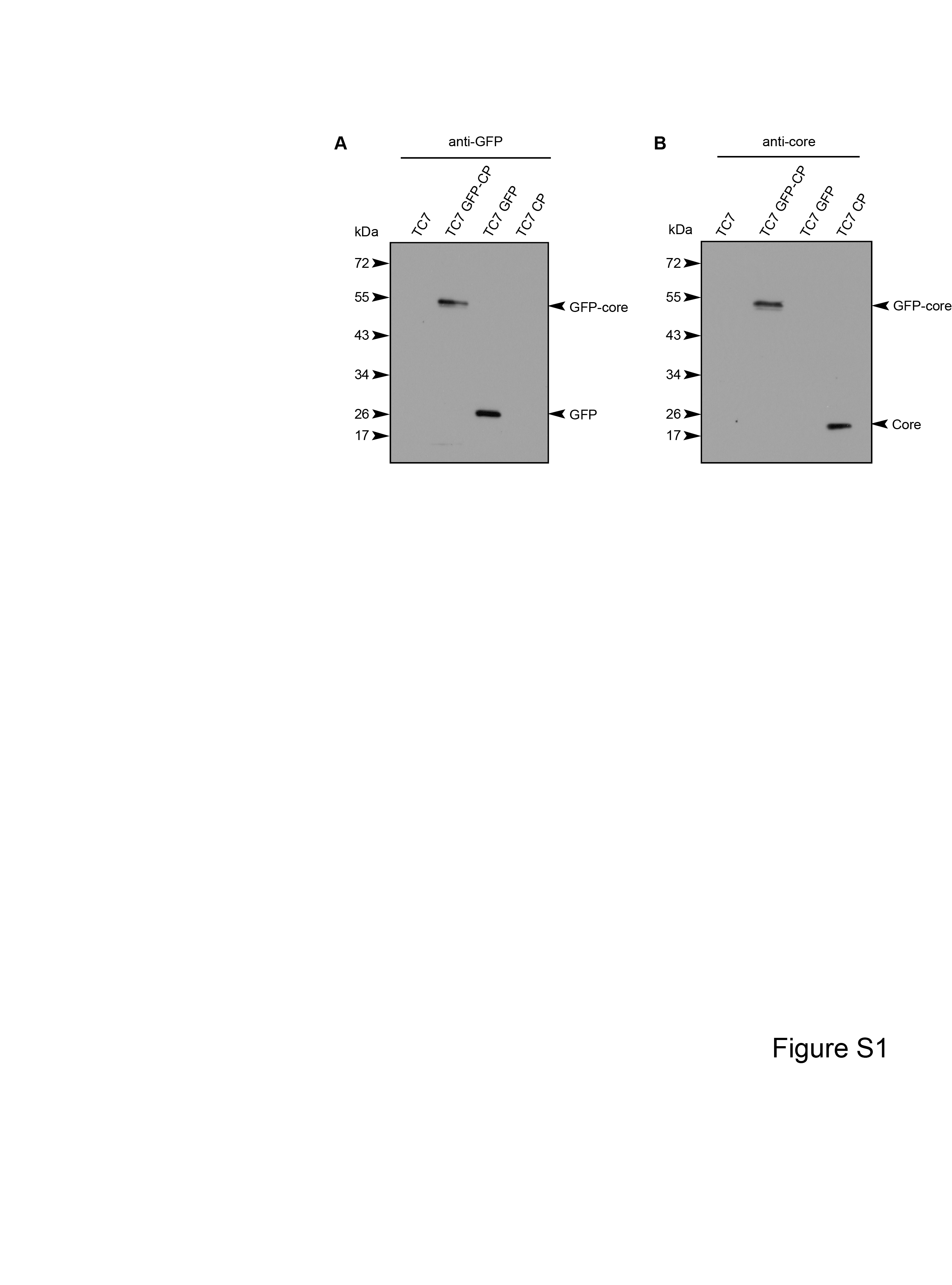

Supplement: Figure S1 — Western blot analysis of GFP-HCV core protein produced by the stably transfected Caco-2/TC7 GFP-CP cell line. Caco-2/TC7 cells expressing GFP-HCV core protein (TC7 GFP-CP) were grown on filters for 18 days then cell lysates were analyzed for GFP (A) or HCV core protein (B). Cell lysates from Caco-2/TC7 cells, Caco-2/TC7 cells transiently transfected with plasmids encoding GFP or HCV core protein are included as controls. (TIF) [file pone.0053017.s001.tif]

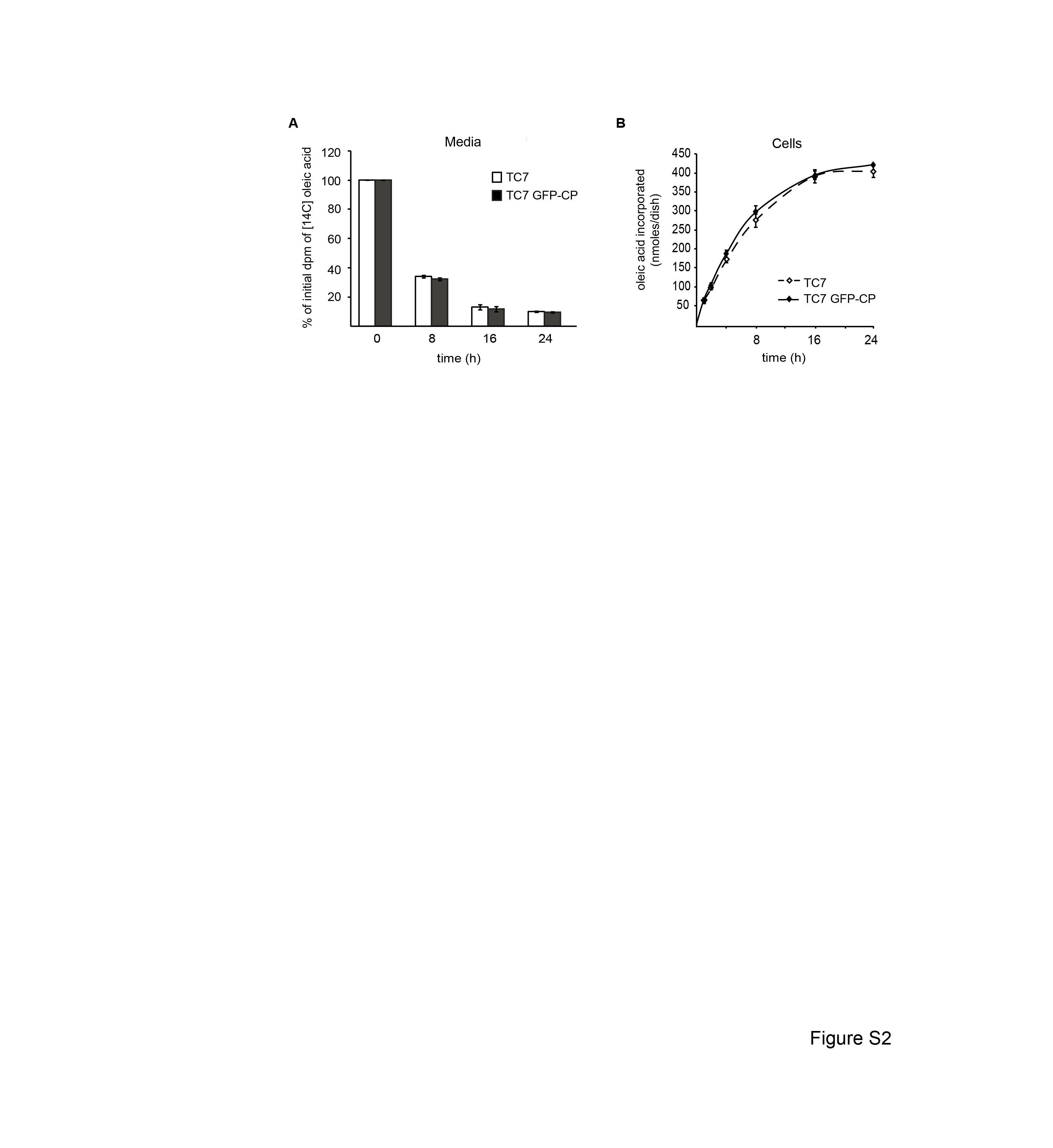

Supplement: Figure S2 — Time course of oleic acid incorporation into Caco-2/TC7 (TC7) and Caco-2/TC7 GFP-CP (TC7 GFP-CP) cells. Cells were grown on filters for 17 days then incubated for various durations with lipid micelles supplemented with [1-14C]oleic acid. The radioactivity remaining in the apical medium was counted and expressed as percentage of the radioactivity present at time 0 (A). Radioactivity contained in cell lysates was counted and expressed as nmoles of oleic acid incorporated per dish (B). Data are means ± SD of three independent experiments performed in duplicate. (TIF) [file pone.0053017.s002.tif]

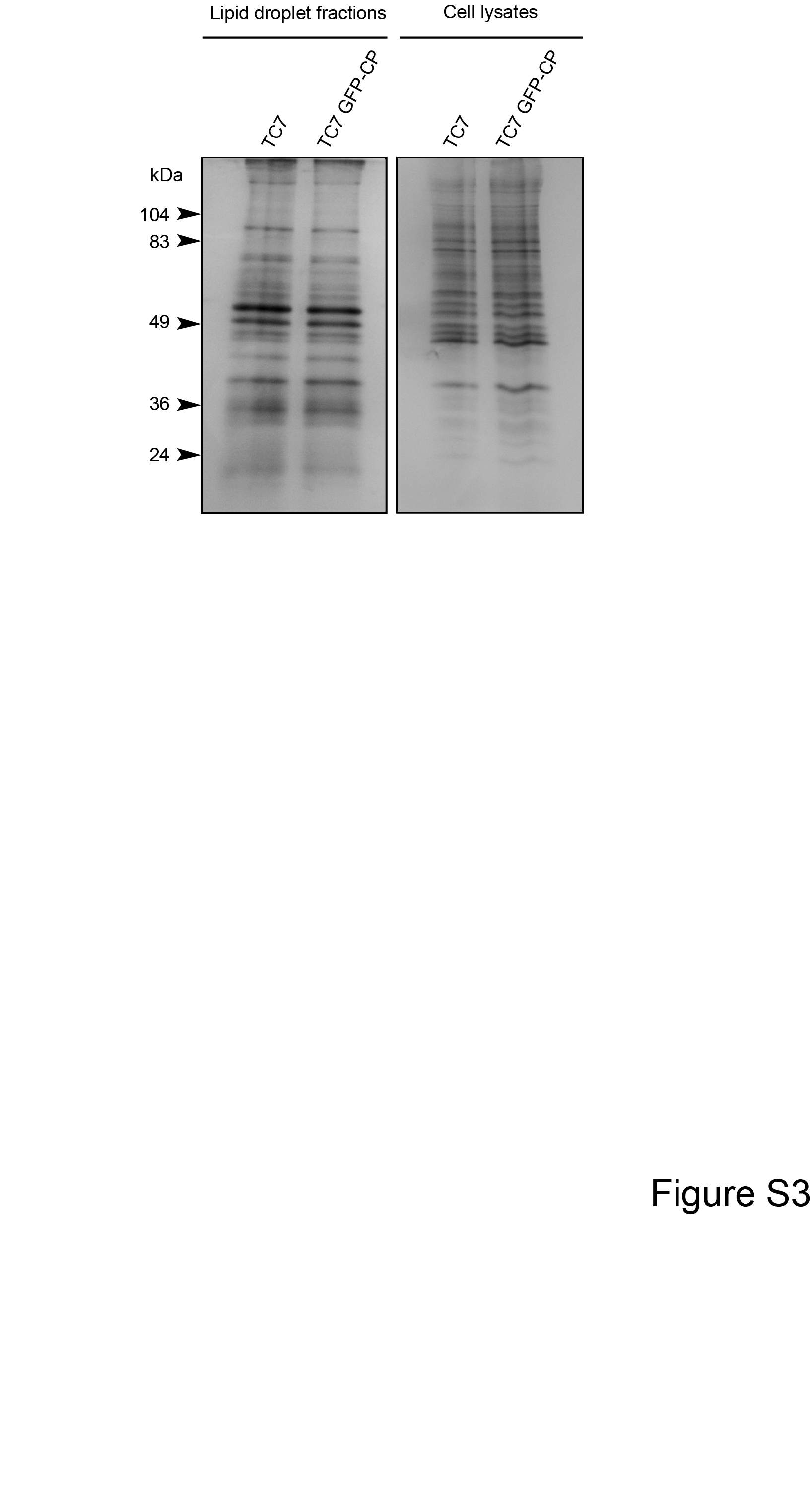

Supplement: Figure S3 — Silver stained gels of lipid droplet fractions and cell lysates from Caco-2/TC7 cells (TC7) and Caco-2/TC7 GFP-CP (TC7 GFP-CP) cells. Cells were cultured on filters for 17 days then supplied with lipid micelles for 24 h. Lipid droplet fractions were prepared as described in the Materials and Methods section, freeze-dried for concentration and one tenth of the lipid droplet fraction was loaded per well. One µg of cell lysates was loaded per well. Proteins were separated by 10% SDS-PAGE and silver stained. (TIF) [file pone.0053017.s003.tif]
