# Supplementary material for: Proteomic Analysis of Lipid Droplets from Caco-2/TC7 Enterocytes Identifies Novel Modulators of Lipid Secretion
Source: PLoS One. 2013 Jan 2;8(1):e53017. doi: 10.1371/journal.pone.0053017 (PMC3534623; doi:10.1371/journal.pone.0053017)
Supplement: Table S1 — Oligonucleotide primers used for gene expression analysis. (DOC) [file pone.0053017.s004.doc]

**Table S1**

**Oligonucleotide primers used for gene expression analysis**

| **Gene** | **Forward Primer** | **Reverse Primer** |
| --- | --- | --- |
| PLIN2 | GTGAGATGGCAGAGAACGGTG | TGCCCCTTTGGTCTTGTCCA |
| HCV core | ATGAGCACGAATCCTAAACC | AGGTTGCGACCGCTCGG |
| HSD17B2 | AGGGAGGCTGGTGAATGTC | CGCCTTTGATGAGCCATAAG |
| MGLL | CGATTTGCCTGGTTCTGATT | GCTGGAAGGGTCTTCAGGT |
| C2orf43 | ATTAGAACCATTCTGCCTTGCT | GCTCCTTTATGGTTTCGTCATC |
| ACSL3 | GGCATCATTGTGCATACCAT | TTGCTATGAGGTTGGTTTTCC |
| LPCAT2 | CTGTCTTGTGCAACCCTTCC | CCTCATCAACGTCAAACAGC |
| HSD3B1 | TCTTCGGTGTCACTCACAGAG | GGCACACTAGCTTGGACACA |
| L19 | AAGATCGATCGCCACATGTAT | TGCGTGCTTCCTTGGTCTTAG |

HCV core, hepatitis C virus core protein; HSD17B2, Estradiol 17-beta-dehydrogenase 2/ 17ß-hydroxysteroid dehydrogenase type 2; MGLL, monoacylglycerol lipase; C2orf43, UPF0554 protein C2orf43; ACSL3, long-chain-fatty-acid--CoA ligase 3; LPCAT2, lysophosphatidylcholine acyltransferase 2; HSD3B1, 3-beta-hydroxysteroid dehydrogenase/Delta 5è4-isomerase type 1; L19, ribosomal protein L19.
